# Supplementary material for: Hybridization promotes asexual reproduction in Caenorhabditis nematodes
Source: PLoS Genet. 2019 Dec 16;15(12):e1008520. doi: 10.1371/journal.pgen.1008520 (PMC6946170; doi:10.1371/journal.pgen.1008520)
Supplement: S1 Fig — (A) Wild isolates of C. becei (QG704 and QG711) and C. nouraguensis (NIC59 and JU1825) have high levels of intra-strain viability. All strains have a sex ratio skewed towards females, some of which show a statistically significant difference from a 50:50 sex ratio (Fisher's exact test with Bonferroni correction, JU1825 p = 1.0, NIC59 p = 0.06, QG711 p = 0.03, QG704 p = 0.03). The total number of offspring quantified for each cross is shown to the right of each bar graph. Data from both graphs are derived from the same crosses. (B) A graph showing the number of embryos laid for intraspecies C. nouraguensis crosses (10 NIC59 females x 10 NIC59 males) and interspecies C. nouraguensis female x C. becei male crosses (10 NIC59 females x 10 QG711 males) in a one-hour window on each of the first three days after the crosses were set. There are three replicates for each type of cross. Each point represents the number of embryos laid for a replicate in a one-hour window that day and the bar graph shows the average of those replicates. The C. nouraguensis female x C. becei male interspecies hybridization had significantly less embryos on days 2 and 3 of egg-laying as compared to the intraspecies C. nouraguensis crosses (*, day 2 p = 0.04, day 3 p = 0.04, Kruskal-Wallis test). (PDF) [file pgen.1008520.s001.pdf]

S1 Fig

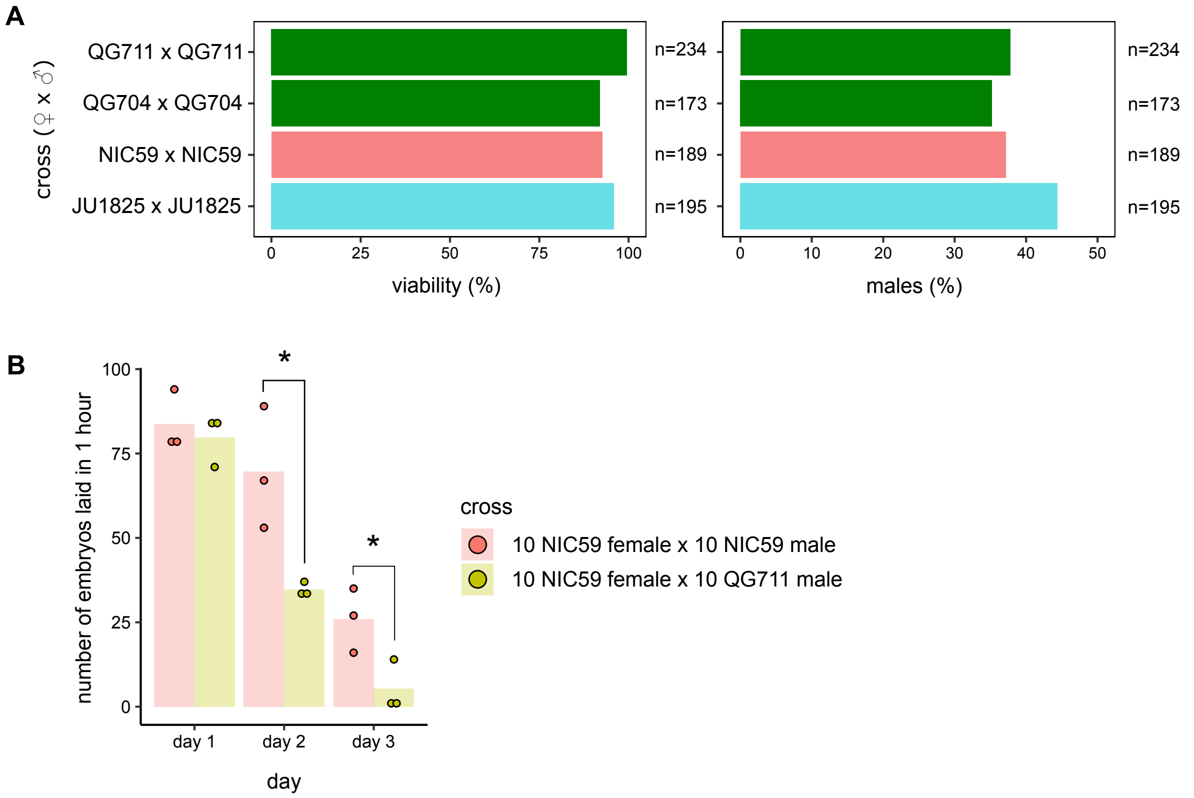

S1 Fig. Crosses measuring intra-strain viability and intraspecies and interspecies fecundity. (A) Wild isolates of *C. becei* (QG704 and QG711) and *C. nouraguensis* (NIC59 and JU1825) have high levels of intra-strain viability. All strains have a sex ratio skewed towards females, some of which show a statistically significant difference from a 50:50 sex ratio (Fisher's exact test with Bonferroni correction, JU1825 p=1.0, NIC59 p=0.06, QG711 p=0.03, QG704 p=0.03). The total number of offspring quantified for each cross is shown to the right of each bar graph. Data from both graphs are derived from the same crosses. **(B)** A graph showing the number of embryos laid for intraspecies *C. nouraguensis* crosses (10 NIC59 females x 10 NIC59 males) and interspecies *C. nouraguensis* female x *C. becei* male crosses (10 NIC59 females x 10 QG711 males) in a one-hour window on each of the first three days after the crosses were set. There are three replicates for each type of cross. Each point represents the number of embryos laid for a replicate in a one-hour window that day and the bar graph shows the average of those replicates. The *C. nouraguensis* female x *C. becei* male interspecies hybridization had significantly less embryos on days 2 and 3 of egg-laying as compared to the intraspecies *C. nouraguensis* crosses (*, day 2 p=0.04, day 3 p=0.04, Kruskal-Wallis test).
