# Supplementary material for: Hybridization promotes asexual reproduction in Caenorhabditis nematodes
Source: PLoS Genet. 2019 Dec 16;15(12):e1008520. doi: 10.1371/journal.pgen.1008520 (PMC6946170; doi:10.1371/journal.pgen.1008520)
Supplement: S2 Fig — Rows show the females of each cross and males are shown in columns. The wild isolate strains used for each species are indicated. Black boxes are intraspecies crosses. Grey boxes are untested interspecies hybridizations. Rare viable F1 adults are present only when crossing C. nouraguensis females to C. becei males. Rare viable but sick F1 larvae are present in both directions of C. becei x C. yunquensis crosses. Worms mate but do not produce F1 embryos in C. panamensis female x C. nouraguensis male and C. becei female x C. panamensis male crosses. At least 12,000 dead F1 were screened for each cross that gave embryos. (PDF) [file pgen.1008520.s002.pdf]

S2 Fig

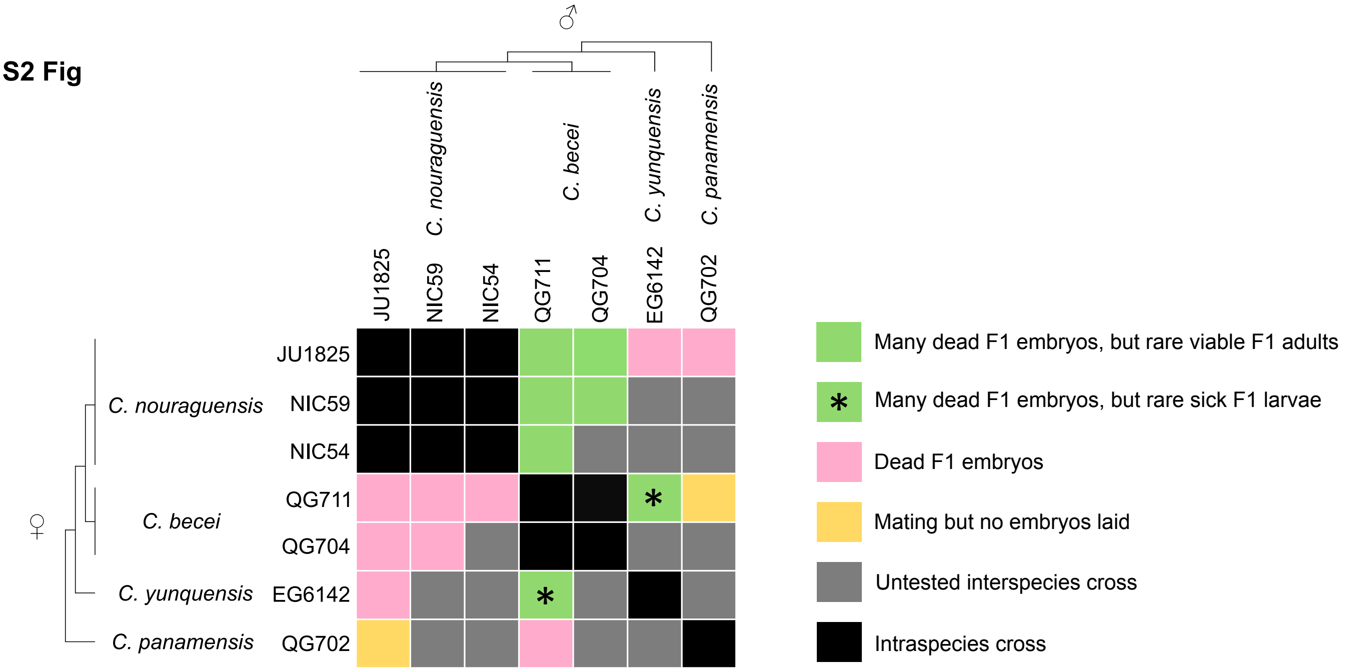

S2 Fig. Summary of interspecies crosses. Rows show the females of each cross while males are shown in columns. The wild isolate strains used for each species are indicated. Black boxes are intraspecies crosses. Grey boxes are untested interspecies hybridizations. Rare viable F1 adults are present only when crossing *C. nouraguensis* females to *C. becei* males. Rare viable but sick F1 larvae are present in both directions of *C. becei* x *C. yunquensis* crosses. Worms mate but do not produce F1 embryos in *C. panamensis* female x *C. nouraguensis* male and *C. becei* female x *C. panamensis* male crosses. At least 12,000 dead F1 were screened for each cross that gave embryos.
