# Supplementary material for: Hybridization promotes asexual reproduction in Caenorhabditis nematodes
Source: PLoS Genet. 2019 Dec 16;15(12):e1008520. doi: 10.1371/journal.pgen.1008520 (PMC6946170; doi:10.1371/journal.pgen.1008520)
Supplement: S3 Fig — (A) We tested the fertility of rare viable F1 derived from C. nouraguensis JU1825 x C. becei QG704 crosses by backcrossing to JU1825 individuals of the opposite sex. F1 were then genotyped at the ITS2 locus using a PCR-RFLP assay. (B) A gel showing the sex, fertility and genotype at the ITS2 locus of viable adult F1. All fertile F1 have a maternal genotype, with one exception: one hybrid F1 female laid inviable F2 embryos (F*, still considered fertile). All sterile F1 had a hybrid genotype. (C) A table summarizing the genotyping data in (B). (PDF) [file pgen.1008520.s003.pdf]

S3 Fig

A

C. nouraguensis (JU1825) ♀ x *C. becei* (QG704) ♂

↓

Rare viable F1

1) Backcross F1 to JU1825 to test fertility (F= fertile, F*=fertile but dead F2 progeny, S=sterile).
2) PCR genotype F1 at ITS2 locus (Primers= 5.8S-1 + 28S-22, HindIII-HF digest).

B

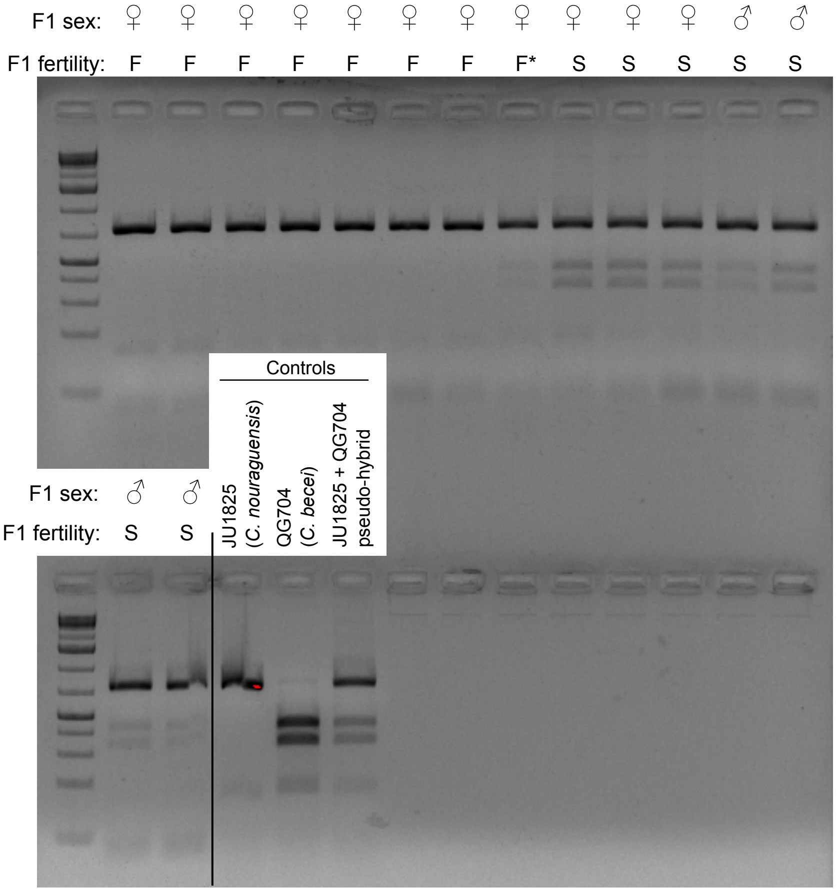

C

Fertility of F1 adult	Hybrid (*C. nouraguensis*/*C. becei*)		Maternal (*C. nouraguensis*)		
	female	male	female	male	total
Fertile	1	0	7	0	8
Sterile	3	4	0	0	7
total	4	4	7	0	15

S3 Fig. Fertile progeny with a maternal genotype and sterile progeny with a hybrid genotype are produced when *C. nouraguensis* females are crossed to males of a different *C. becei* strain. (A) We tested the fertility of rare viable F1 derived from *C. nouraguensis* JU1825 x *C. becei* QG704 crosses by backcrossing to JU1825 individuals of the opposite sex. F1 were then genotyped at the ITS2 locus using a PCR-RFLP assay. **(B)** A gel showing the sex, fertility and genotype at the ITS2 locus of viable adult F1. All fertile F1 have a maternal genotype, with one exception: one hybrid F1 female laid inviable F2 embryos (F*, still considered fertile). All sterile F1 had a hybrid genotype. **(C)** A table summarizing the genotyping data in (B).
