# Supplementary material for: Hybridization promotes asexual reproduction in Caenorhabditis nematodes
Source: PLoS Genet. 2019 Dec 16;15(12):e1008520. doi: 10.1371/journal.pgen.1008520 (PMC6946170; doi:10.1371/journal.pgen.1008520)
Supplement: S4 Fig — The -1 oocytes from C. nouraguensis JU1825 and C. becei QG711 females primarily have six DAPI- staining bodies (two examples shown here). Most fertile F1 females derived from JU1825 female x QG711 male crosses have six DAPI-staining bodies (Example fertile F1 female #1). A minority have eight DAPI-staining bodies. In three of these cases, there appear to be seven relatively normal sized DAPI bodies plus a very small one (Example fertile F1 female #2, small DAPI body highlighted by white arrowhead). In the other two cases, all eight DAPI bodies appear roughly equal in size (Example fertile F1 female #3). This higher number of DAPI-staining bodies is not the chance observation of a low frequency meiotic defect in a nucleus that happens to be in the -1 oocyte position (for example, homologs fail to recombine and increase the number of univalents) because we observed the same number of DAPI-staining bodies in both germlines of the same fertile F1 female. We hypothesize that these extra DAPI-staining bodies represent extra DNA (either maternal or paternal) in addition to the two chromatids inherited from each maternal bivalent. Scale bar: 5 μm. (PDF) [file pgen.1008520.s004.pdf]

S4 Fig

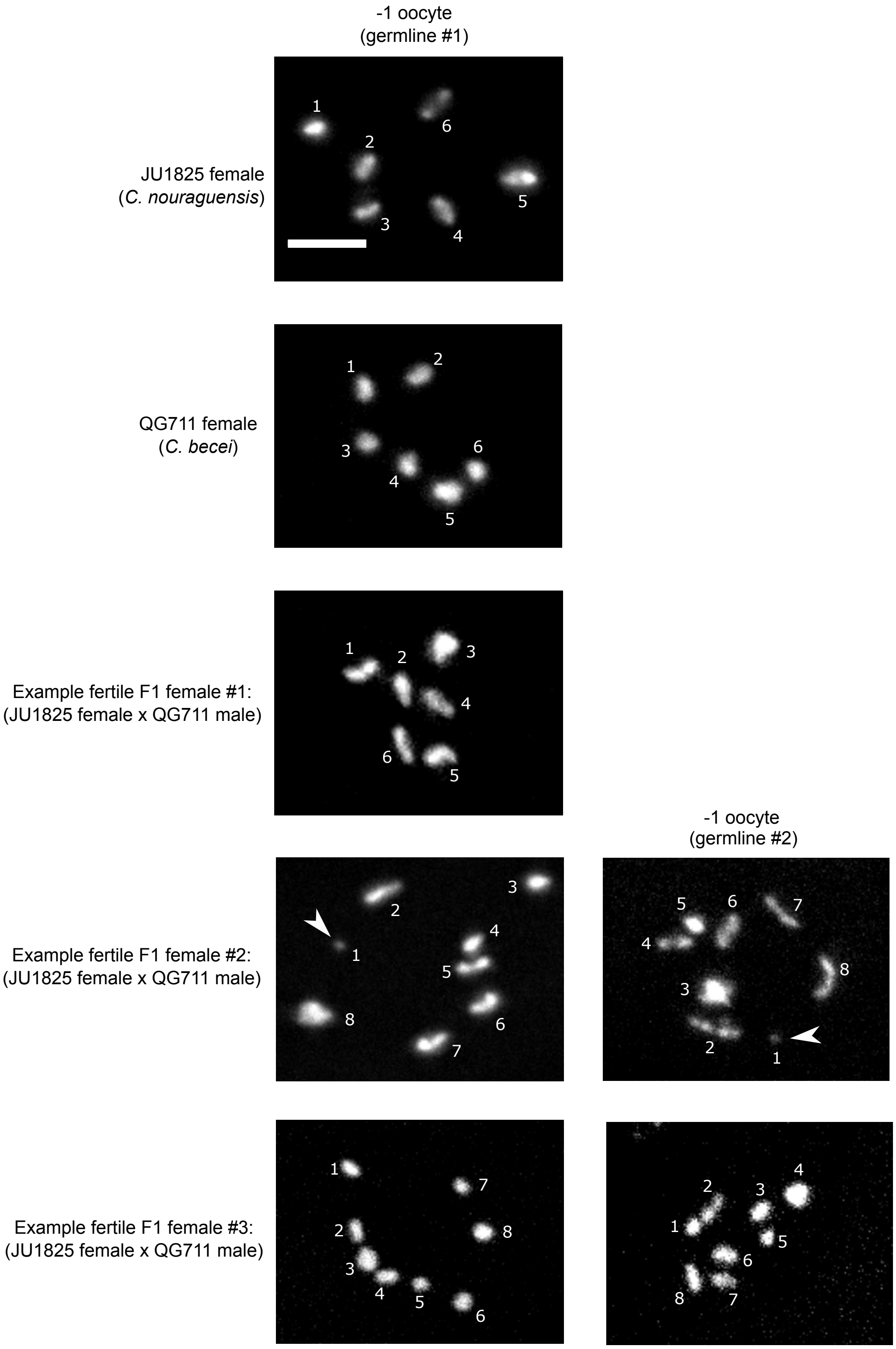

S4 Fig. Fertile F1 females are diploid. The -1 oocytes from *C. nouraguensis* (JU1825) and *C. becei* (QG711) females primarily have six DAPI-staining bodies (two examples shown here). Most fertile F1 females derived from JU1825 female x QG711 male crosses have six DAPI-staining bodies (Example fertile F1 female #1). A minority have eight DAPI-staining bodies. In three of these cases, there appear to be seven relatively normal sized DAPI bodies plus a very small one (Example fertile F1 female #2, small DAPI body highlighted by white arrowhead). In the other two cases, all eight DAPI bodies appear roughly equal in size (Example fertile F1 female #3). This higher number of DAPI-staining bodies is not the chance observation of a low frequency meiotic defect in a nucleus that happens to be in the -1 oocyte position (for example, homologs fail to recombine and increase the number of univalents) because we observed the same number of DAPI-staining bodies in both germlines of the same fertile F1 female. We hypothesize that these extra DAPI-staining bodies represent extra DNA (either maternal or paternal) in addition to the two chromatids inherited from each maternal bivalent. Scale bar: 5 µm.
