# Supplementary material for: Hybridization promotes asexual reproduction in Caenorhabditis nematodes
Source: PLoS Genet. 2019 Dec 16;15(12):e1008520. doi: 10.1371/journal.pgen.1008520 (PMC6946170; doi:10.1371/journal.pgen.1008520)
Supplement: S5 Fig — (A) The six possible ways of combining two of the four genetically distinct chromosomes in a bivalent are illustrated, with their expected genotypic signature below them. There are five distinct genotypic signatures. Two result from combining sister-chromatids and are called "Sisters_1" and "Sisters_2". Three result from combining homologous chromosomes and are called "Homologs_1", "Homologs_2" and "Homologs_3". (B) A table summarizing the genotype of each maternal chromosome for each sequenced F1 individual. The F1's sex and fertility are noted. Each individual’s ploidy is inferred from the genotyping data. The genotype "Homologs_ambiguous" refers to chromosomes that are heterozygous in their centers, but one end of the chromosome is not obviously heterozygous (N/J) or homozygous for either NIC59 or JU1825. The genotype "Hemizygous X" refers to hemizygous X-chromosomes in males that have half the read depth of the autosomes. The genotype "Triploid" refers to chromosomes that have three copies instead of two based on relative read depth and whole-chromosome genotype. The “Sisters_3” genotype refers to chromosomes that have inherited two non-recombinant JU1825 chromatids. Three fertile females (F1_8, F1_11 and F1_39) have NIC59 and JU1825 alleles in the center of their chromosomes, but exhibit a slight skew from the expected 0.50 NIC59 allele frequency (S7 Fig). We hypothesize that this skew is due to contaminating DNA derived from backcrossing each female when testing her fertility. For example, a viable female that was backcrossed to a JU1825 male would carry JU1825 sperm and therefore JU1825 DNA in her spermatheca. In this case, the contaminating DNA would skew the female’s entire genome to a lower NIC59 SNP frequency. Consistent with this, in all three females the more abundant allele matches the genotype of the male she was backcrossed to (S1 Table). Correcting for this potential backcrossing contamination, the genotypes of all the chromosomes in these three fem [file pgen.1008520.s005.pdf]

S5 Fig

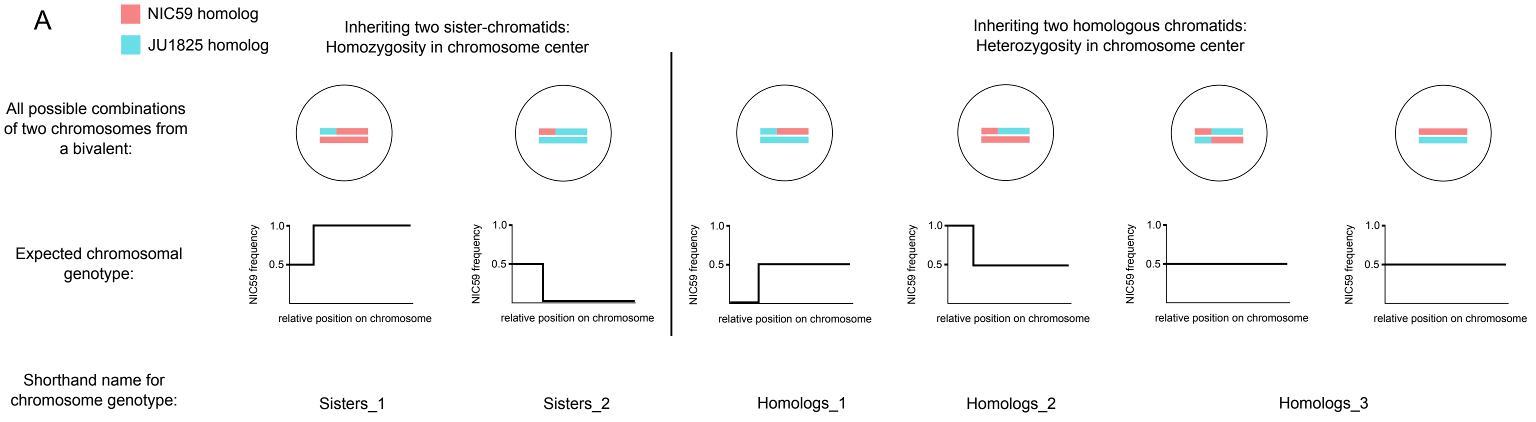

B

				C. nouraguensis chromosome genotypes					
F1 sample name	fertility	sex	inferred ploidy	Chr. I	Chr. II	Chr. III	Chr. IV	Chr. V	Chr. X
F1_1	fertile	female	diploid	Homologs_3	Homologs_3	Homologs_3	Homologs_2	Homologs_2	Homologs_2
F1_5	fertile	female	diploid	Homologs_2	Homologs_3	Homologs_3	Homologs_1	Homologs_2	Homologs_1
F1_29	fertile	female	diploid	Homologs_3	Homologs_ambiguous	Homologs_3	Homologs_1	Homologs_3	Homologs_3
F1_41	fertile	female	diploid	Homologs_2	Homologs_3	Homologs_3	Homologs_3	Homologs_3	Homologs_3
F1_8	fertile	female	likely diploid, backcross contamination	Homologs_3	Homologs_3	Homologs_3	Homologs_3	Homologs_3	Homologs_2
F1_11	fertile	female	likely diploid, backcross contamination	Homologs_3	Homologs_3	Homologs_3	Homologs_1	Homologs_1	Homologs_3
F1_39	fertile	female	likely diploid, backcross contamination	Homologs_3	Homologs_1	Homologs_3	Homologs_1	Homologs_3	Homologs_1
F1_25	fertile	female	ambiguous, backcross contamination	ambiguous	ambiguous	ambiguous	ambiguous	ambiguous	ambiguous
F1_46	fertile	male	diploid	Homologs_ambiguous	Homologs_3	Homologs_2	Homologs_1	Homologs_3	Hemizygous X
F1_4	fertile	male	diploid	Homologs_2	Homologs_3	Homologs_3	Triploid	Homologs_3	Hemizygous X
F1_48	fertile	male	diploid	Homologs_2	Homologs_3	Triploid	Homologs_1	Homologs_3	Hemizygous X
F1_26	sterile	female	ambiguous, backcross contamination	ambiguous	ambiguous	ambiguous	ambiguous	ambiguous	ambiguous
F1_18	sterile	female	ambiguous, backcross contamination	ambiguous	ambiguous	ambiguous	ambiguous	ambiguous	ambiguous
F1_6	sterile	female	triploid hybrid	Homologs_2	Homologs_3	Homologs_3	Homologs_3	Homologs_1	Homologs_1
F1_12	sterile	female	triploid hybrid	Homologs_1	Homologs_ambiguous	Homologs_3	Homologs_3	Homologs_3	Homologs_3
F1_20	sterile	female	triploid hybrid	Homologs_2	Homologs_2	Homologs_3	Homologs_3	Homologs_3	Homologs_1
F1_16	sterile	male	diploid-triploid hybrid	Homologs_1	Homologs_1	Homologs_1	Homologs_3	Homologs_3	Hemizygous X
F1_23	sterile	male	diploid-triploid hybrid	Homologs_3	Homologs_2	Homologs_1	Homologs_1	Sisters_3	Hemizygous X
F1_10	sterile	male	diploid-triploid hybrid	Homologs_2	Homologs_2	Homologs_3	Triploid	Homologs_3	Homologs_2
F1_17	sterile	male	triploid hybrid	Homologs_3	Homologs_3	Sisters_1	Homologs_2	Homologs_2	Homologs_2
F1_21	sterile	male	triploid hybrid	Homologs_3	Homologs_1	Homologs_1	Homologs_2	Homologs_2	Homologs_2

C

Fertile F1

Chromosome genotype	Chr. I	Chr. II	Chr. III	Chr. IV	Chr. V	Chr. X	total
Homologs_1	0	1	0	6	1	2	10
Homologs_2	4	0	1	1	2	2	10
Homologs_3	5	8	8	2	7	3	33
Homologs_ambiguous	1	1	0	0	0	0	2
Sisters_1	0	0	0	0	0	0	0
Sisters_2	0	0	0	0	0	0	0
Sisters_3	0	0	0	0	0	0	0
Hemizygous X	0	0	0	0	0	3	3
Triploid	0	0	1	1	0	0	2
ambiguous	1	1	1	1	1	1	6
total	11	11	11	11	11	11	66

Sterile F1

Chromosome genotype	Chr. I	Chr. II	Chr. III	Chr. IV	Chr. V	Chr. X	total
Homologs_1	2	2	3	1	1	2	11
Homologs_2	3	3	0	2	2	3	13
Homologs_3	3	2	4	4	4	1	18
Homologs_ambiguous	0	1	0	0	0	0	1
Sisters_1	0	0	1	0	0	0	1
Sisters_2	0	0	0	0	0	0	0
Sisters_3	0	0	0	0	1	0	1
Hemizygous X	0	0	0	0	0	2	2
Triploid	0	0	0	1	0	0	1
ambiguous	2	2	2	2	2	2	12
total	10	10	10	10	10	10	60

Fertile and Sterile F1

Chromosome genotype	Chr. I	Chr. II	Chr. III	Chr. IV	Chr. V	Chr. X	total
Homologs_1	2	3	3	7	2	4	21
Homologs_2	7	3	1	3	4	5	23
Homologs_3	8	10	12	6	11	4	51
Homologs_ambiguous	1	2	0	0	0	0	3
Sisters_1	0	0	1	0	0	0	1
Sisters_2	0	0	0	0	0	0	0
Sisters_3	0	0	0	0	1	0	1
Hemizygous X	0	0	0	0	0	5	5
Triploid	0	0	1	2	0	0	3
ambiguous	3	3	3	3	3	3	18
total	21	21	21	21	21	21	126

S5 Fig. Fertile F1 inherit two randomly selected homologous chromatids from each maternal bivalent. (A) The six possible ways of combining two of the four genetically distinct chromosomes in a bivalent are illustrated, with their expected genotypic signature below them. There are five distinct genotypic signatures. Two result from combining sister-chromatids and are called "Sisters_1" and "Sisters_2". Three result from combining homologous chromosomes and are called "Homologs_1", "Homologs_2" and "Homologs_3". **(B)** A table summarizing the genotype of each maternal chromosome for each sequenced F1 individual. The F1's sex and fertility are noted. Each individual's ploidy is inferred from the genotyping data. The genotype "Homologs_ambiguous" refers to chromosomes that are heterozygous in their centers, but one end of the chromosome is not obviously heterozygous (N/J) or homozygous for either NIC59 or JU1825. The genotype "Hemizygous X" refers to hemizygous X-chromosomes in males that have half the read depth of the autosomes. The genotype "Triploid" refers to chromosomes that have three copies instead of two based on relative read depth and whole chromosome genotype. The "Sisters_3" genotype refers to chromosomes that have inherited two non-recombinant JU1825 chromatids. Three fertile females (F1_8, F1_11 and F1_39) have NIC59 and JU1825 alleles in the center of their chromosomes, but exhibit a slight skew from the expected 0.50 NIC59 allele frequency (S7 Fig). We hypothesize that this skew is due to contaminating DNA derived from backcrossing each female when testing her fertility. For example, a viable female that was backcrossed to a JU1825 male would carry JU1825 sperm and therefore JU1825 DNA in her spermatheca. In this case, the contaminating DNA would skew the female's entire genome to a lower NIC59 SNP frequency. Consistent with this, in all three females the more abundant allele matches the genotype of the male she was backcrossed to (S1 Table). Correcting for this potential backcrossing contamination, the genotypes of all the chromosomes in these three females are consistent with the inheritance of two homologous chromatids. One fertile female (F1_25) and two sterile females (F1_18 and F1_26) have a very low proportion of NIC59 reads across their entire genome (S7 Fig). We hypothesize this skew is due to contaminating DNA from JU1825 males during fertility testing and poor lysis of the female (S1 Table). The low proportion of NIC59 reads made it difficult to infer actual maternal chromosome genotypes and are therefore categorized as "ambiguous". **(C)** Tables summarizing the frequency of genotypes per chromosome in all fertile F1, all sterile F1, or a combination of all fertile and sterile F1.
