# Supplementary material for: Hybridization promotes asexual reproduction in Caenorhabditis nematodes
Source: PLoS Genet. 2019 Dec 16;15(12):e1008520. doi: 10.1371/journal.pgen.1008520 (PMC6946170; doi:10.1371/journal.pgen.1008520)
Supplement: S6 Fig — (A) A schematic of the cross used to determine how diploidy is restored in gynogenetically produced offspring in the interspecies cross. Heterozygous (NIC59/JU1825) C. nouraguensis females are crossed to C. becei (QG711) males. Individual viable offspring resulting from the hybridization undergo whole-genome sequencing. (B) If diploid maternal inheritance results from mitotic (apomixis) rather than meiotic divisions, then viable F1 are clones of their mother and will be heterozygous NIC59/JU1825 across their entire genome (0.5 NIC59 allele frequency). (C) Diploid maternal inheritance can occur through endomitosis, which is when a haploid maternal genome replicates without a cell division. This results in two exact copies of each chromosome and therefore homozygosity for either NIC59 (1.0 NIC59 allele frequency) or JU1825 alleles (0.0 NIC59 allele frequency) across the entire genome. (D) Diploid maternal inheritance can occur through automixis (combining two of the four meiotic products). Combining two homologous chromatids will result in heterozygosity in the center of a chromosome, and heterozygosity or homozygosity at the chromosome ends. Combining two sister chromatids will result in homozygosity in the center of a chromosome and heterozygosity at one of the ends. Inheriting two sister chromatids for one chromosome and two homologous chromatids for another chromosome in the same genome is theoretically possible, but not depicted here. (PDF) [file pgen.1008520.s006.pdf]

S6 Fig

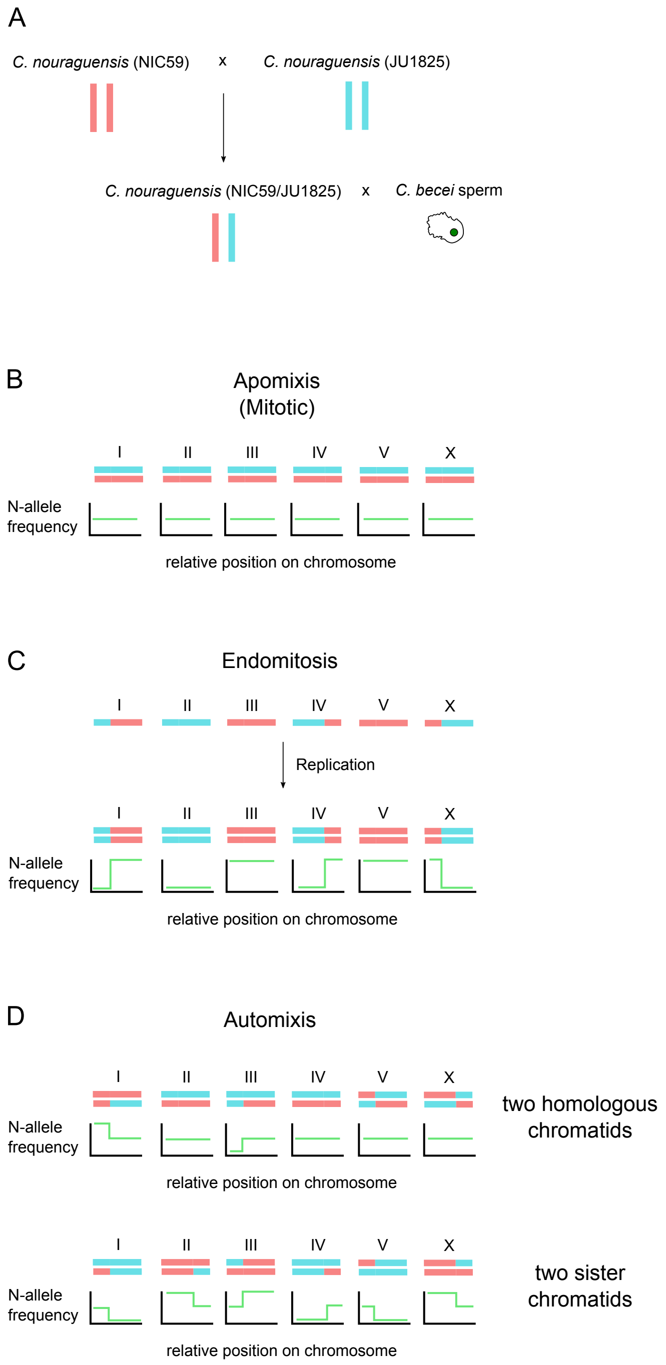

S6 Fig. Genotypic signatures of multiple potential mechanisms of diploid maternal inheritance. (A) A schematic of the cross used to determine how diploidy is restored in gynogenetically produced offspring in the interspecies cross. Heterozygous (NIC59/JU1825) *C. nouraguensis* females are crossed to *C. becei* (QG711) males. Individual viable offspring resulting from the hybridization undergo whole genome sequencing. **(B)** If diploid maternal inheritance results from mitotic (apomixis) rather than meiotic divisions, then viable F1 are clones of their mother and will be heterozygous NIC59/JU1825 across their entire genome (0.5 NIC59 allele frequency). **(C)** Diploid maternal inheritance can occur through endomitosis, which is when a haploid maternal genome replicates without a cell-division. This results in two exact copies of each chromosome and therefore homozygosity for either NIC59 (1.0 NIC59 allele frequency) or JU1825 alleles (0.0 NIC59 allele frequency) across the entire genome. **(D)** Diploid maternal inheritance can occur through automixis (combining two of the four meiotic products). Combining two homologous chromatids will result in heterozygosity in the center of a chromosome, and heterozygosity or homozygosity at the chromosome ends. Combining two sister chromatids will result in homozygosity in the center of a chromosome and heterozygosity at one of their ends. Inheriting two sister chromatids for one chromosome and two homologous chromatids for another chromosome in the same genome is theoretically possible, but not depicted here.
