# Supplementary material for: Hybridization promotes asexual reproduction in Caenorhabditis nematodes
Source: PLoS Genet. 2019 Dec 16;15(12):e1008520. doi: 10.1371/journal.pgen.1008520 (PMC6946170; doi:10.1371/journal.pgen.1008520)
Supplement: S7 Fig — Each of the following pages contains plots describing whole-genome sequencing data of either a rare viable F1 individual or a control DNA sample. The sample name is at the top of each page, along with the individual's sex, fertility and strain it was backcrossed to for fertility testing (if applicable). Each page has five rows of plots. The first row shows the genotypes of the sample's C. nouraguensis maternal chromosomes (i.e. average NIC59 allele frequency in 50-kb windows across the C. nouraguensis assembly). Haplotype change points and average allele frequency for each segment are shown by the green horizontal lines. The second and third rows show the sample's average read coverage of the C. becei and C. nouraguensis assemblies in 50-kb windows. The fourth and fifth rows show the average GC content of the C. becei and C. nouraguensis assemblies in 50-kb windows. The gray vertical lines represent breaks between scaffolds. (PDF) [file pgen.1008520.s007.pdf]

S7 Fig. Fertile F1 inherit two randomly selected homologous chromatids from each maternal bivalent. Each of the following pages contains plots describing whole-genome sequencing data of either a rare viable F1 individual or a control DNA sample. The sample name is at the top of each page, along with the individual's sex, fertility and strain it was backcrossed to for fertility testing (if applicable). Each page has five rows of plots. The first row shows the genotypes of the sample's *C. nouraguensis* maternal chromosomes (i.e. average NIC59 allele frequency in 50-kb windows across the *C. nouraguensis* assembly). Haplotype change points and average allele frequency for each segment are shown by the green horizontal lines. The second and third rows show the sample's average read coverage of the *C. becei* and *C. nouraguensis* assemblies in 50-kb windows. The fourth and fifth rows show the average GC content of the *C. becei* and *C. nouraguensis* assemblies in 50-kb windows. The gray vertical lines represent breaks between scaffolds.

F1_1

sex=female, fert=fertile, matedTo=NIC59 male

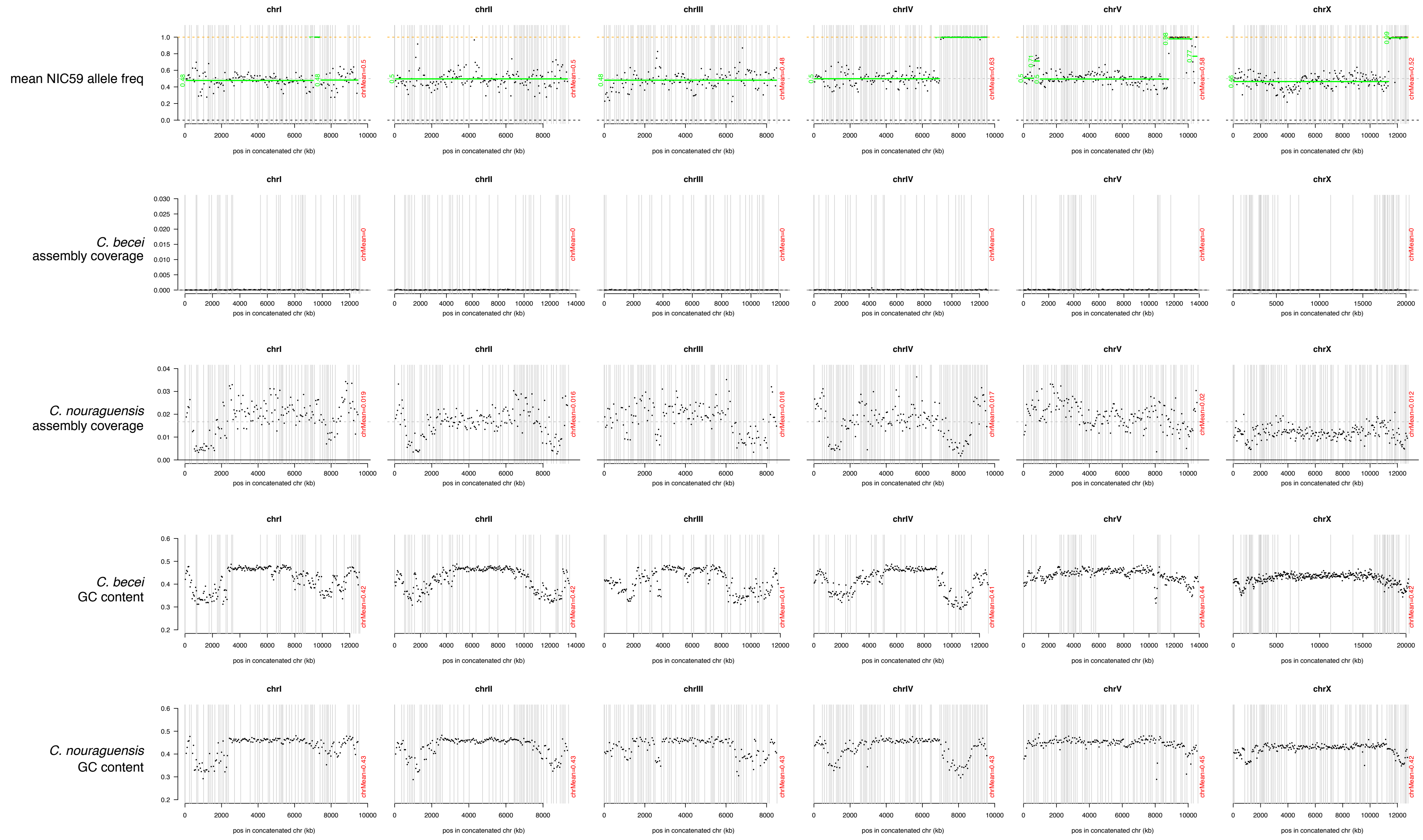

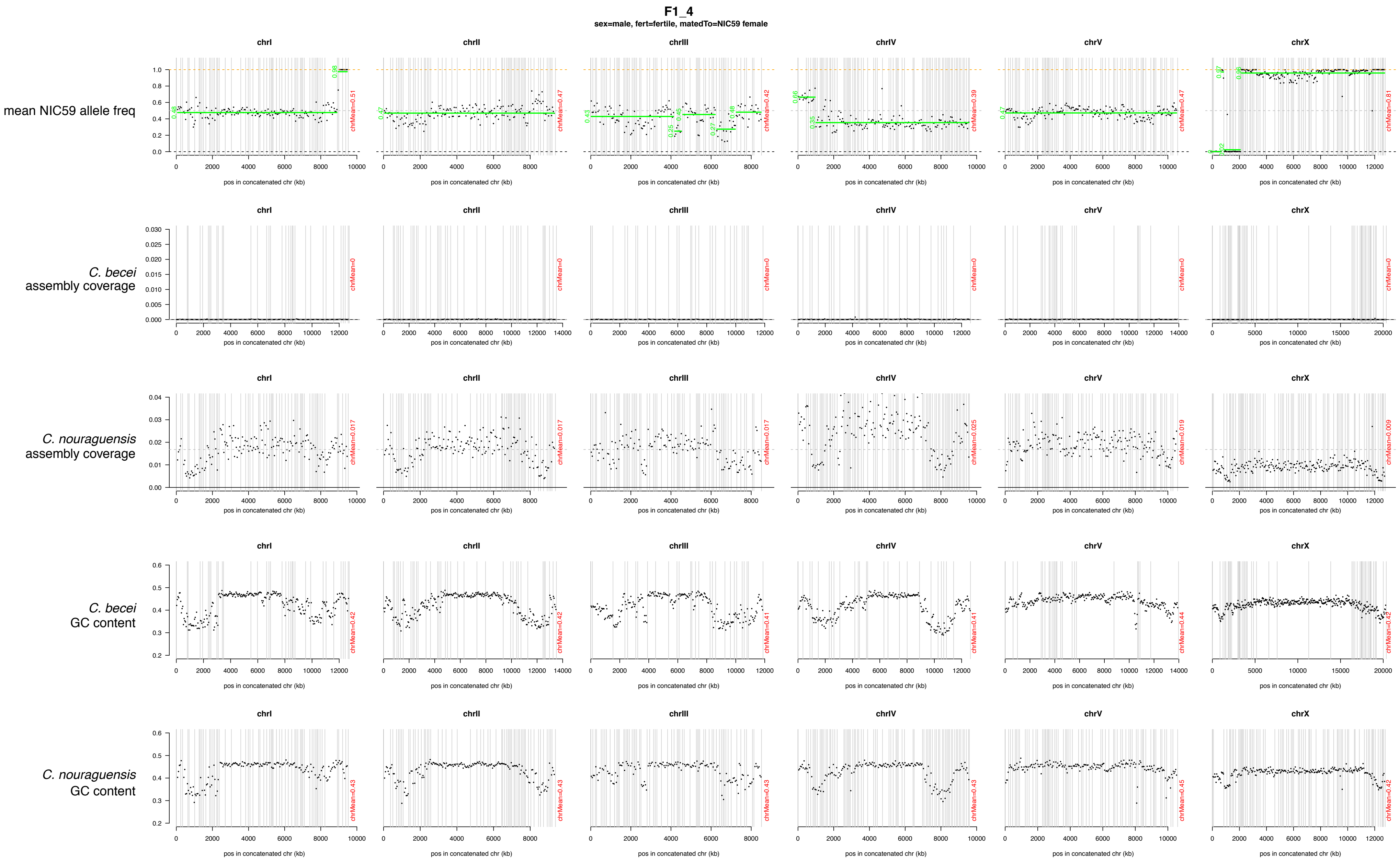
F1_4
sex=male, fert=fertile, matedTo=NIC59 female
mean NIC59 allele freq
C. becei assembly coverage
C. nouraguensis assembly coverage
C. becei GC content
C. nouraguensis GC content
chrI
chrII
chrIII
chrIV
chrV
chrX
pos in concatenated chr (kb)

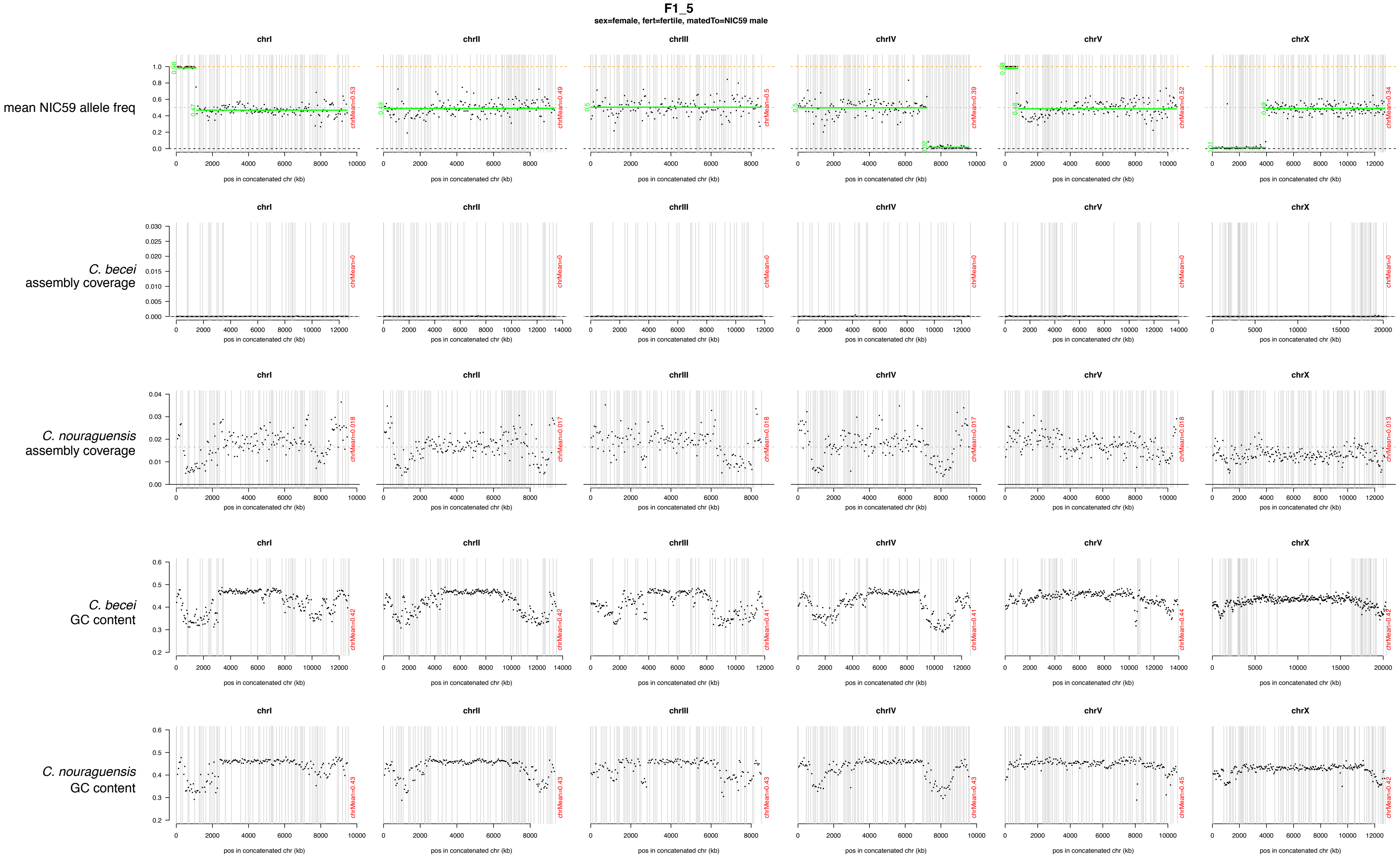
F1_5
sex=female, fert=fertile, matedTo=NIC59 male
chrI
chrII
chrIII
chrIV
chrV
chrX
mean NIC59 allele freq
C. becei assembly coverage
C. nouraguensis assembly coverage
C. becei GC content
C. nouraguensis GC content
pos in concatenated chr (kb)

F1_6

sex=female, fert=sterile, matedTo=NIC59 male

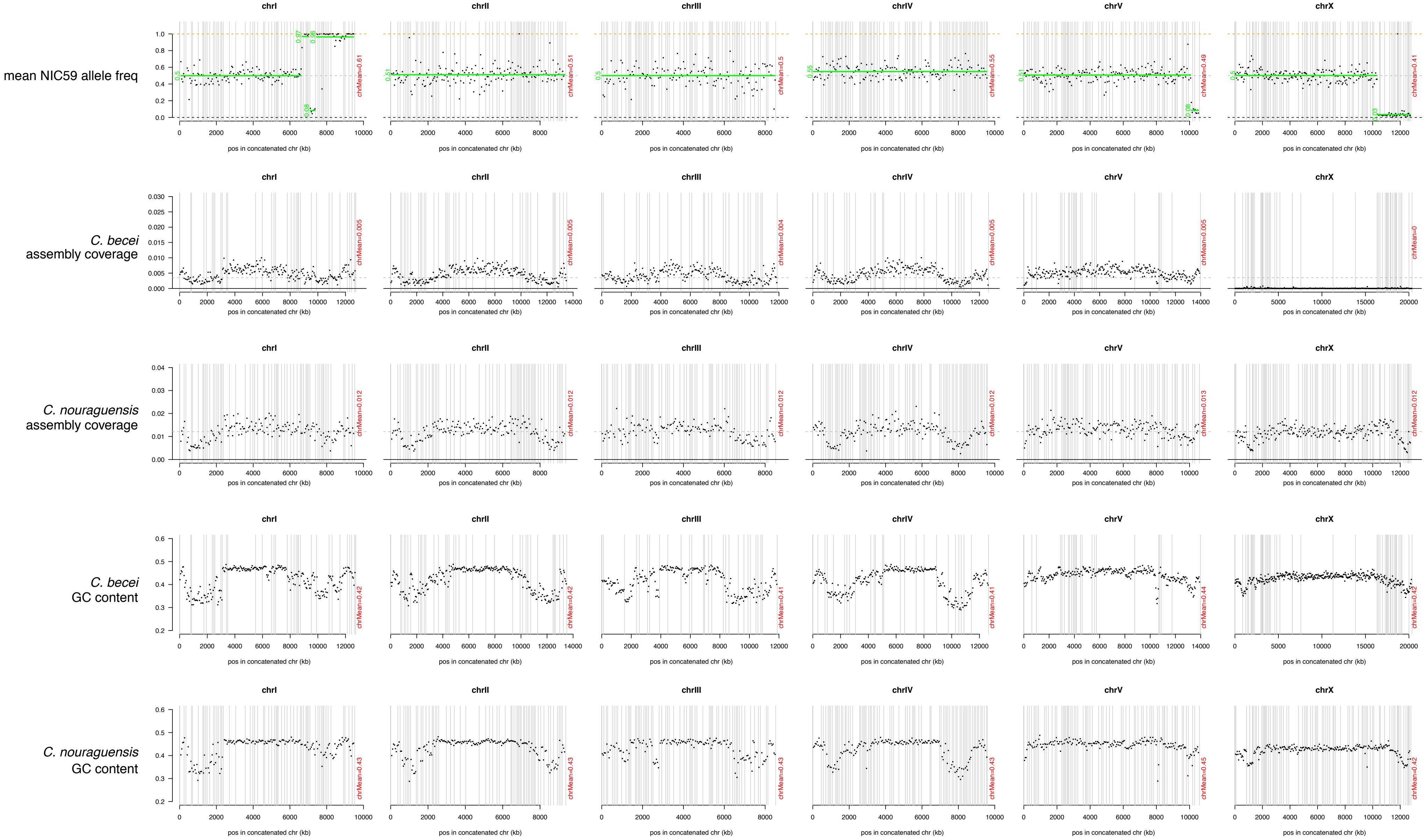

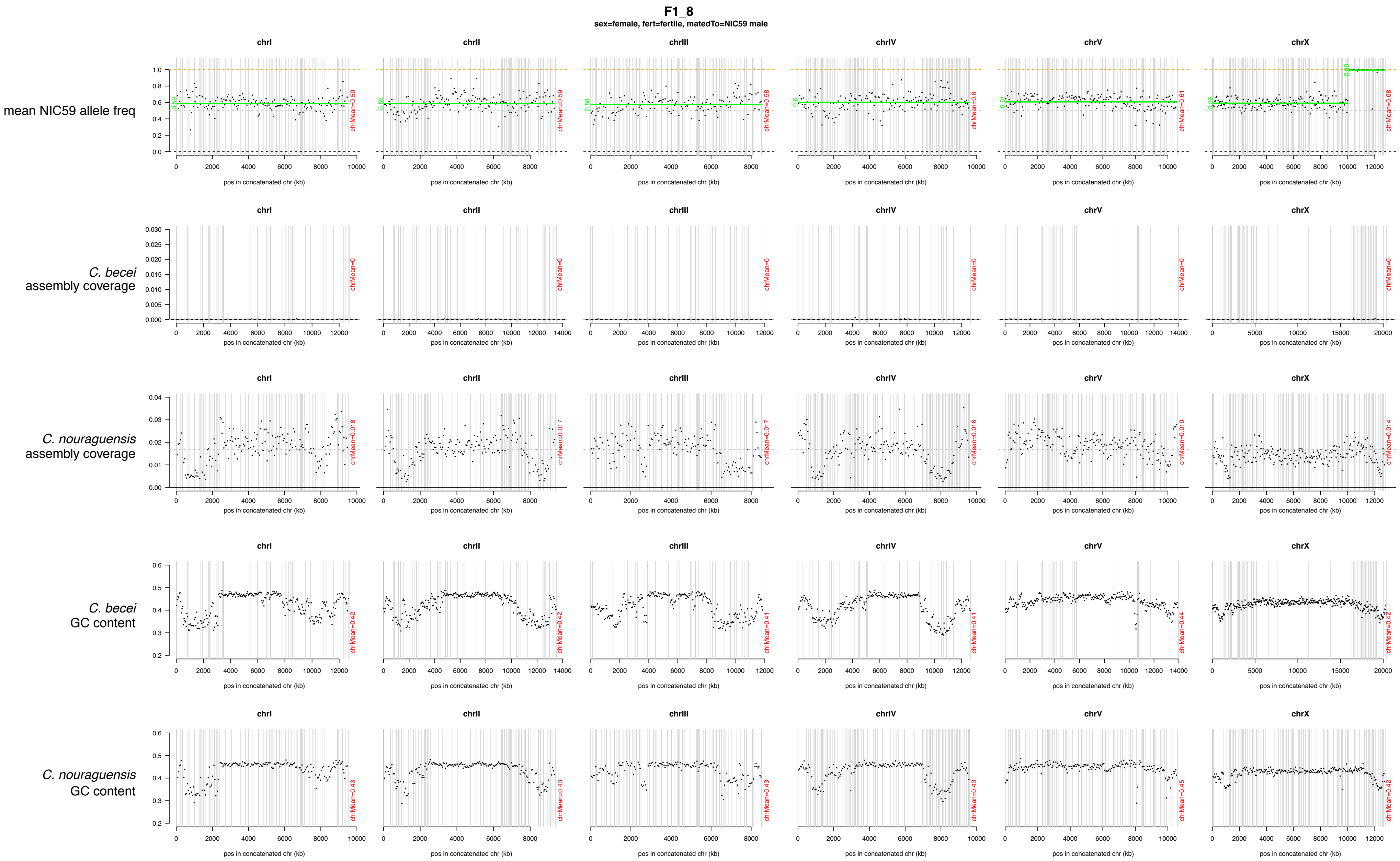
F1_8
sex=female, fert=fertile, matedTo=NIC59 male
chrI
chrII
chrIII
chrIV
chrV
chrX
mean NIC59 allele freq
C. becei assembly coverage
C. nouraguensis assembly coverage
C. becei GC content
C. nouraguensis GC content
pos in concatenated chr (kb)
chrMean=0.59
chrMean=0.59
chrMean=0.58
chrMean=0.6
chrMean=0.61
chrMean=0.68
chrMean=0
chrMean=0.018
chrMean=0.017
chrMean=0.017
chrMean=0.016
chrMean=0.019
chrMean=0.014
chrMean=0.42
chrMean=0.42
chrMean=0.41
chrMean=0.41
chrMean=0.44
chrMean=0.42
chrMean=0.43
chrMean=0.43
chrMean=0.43
chrMean=0.43
chrMean=0.45
chrMean=0.42

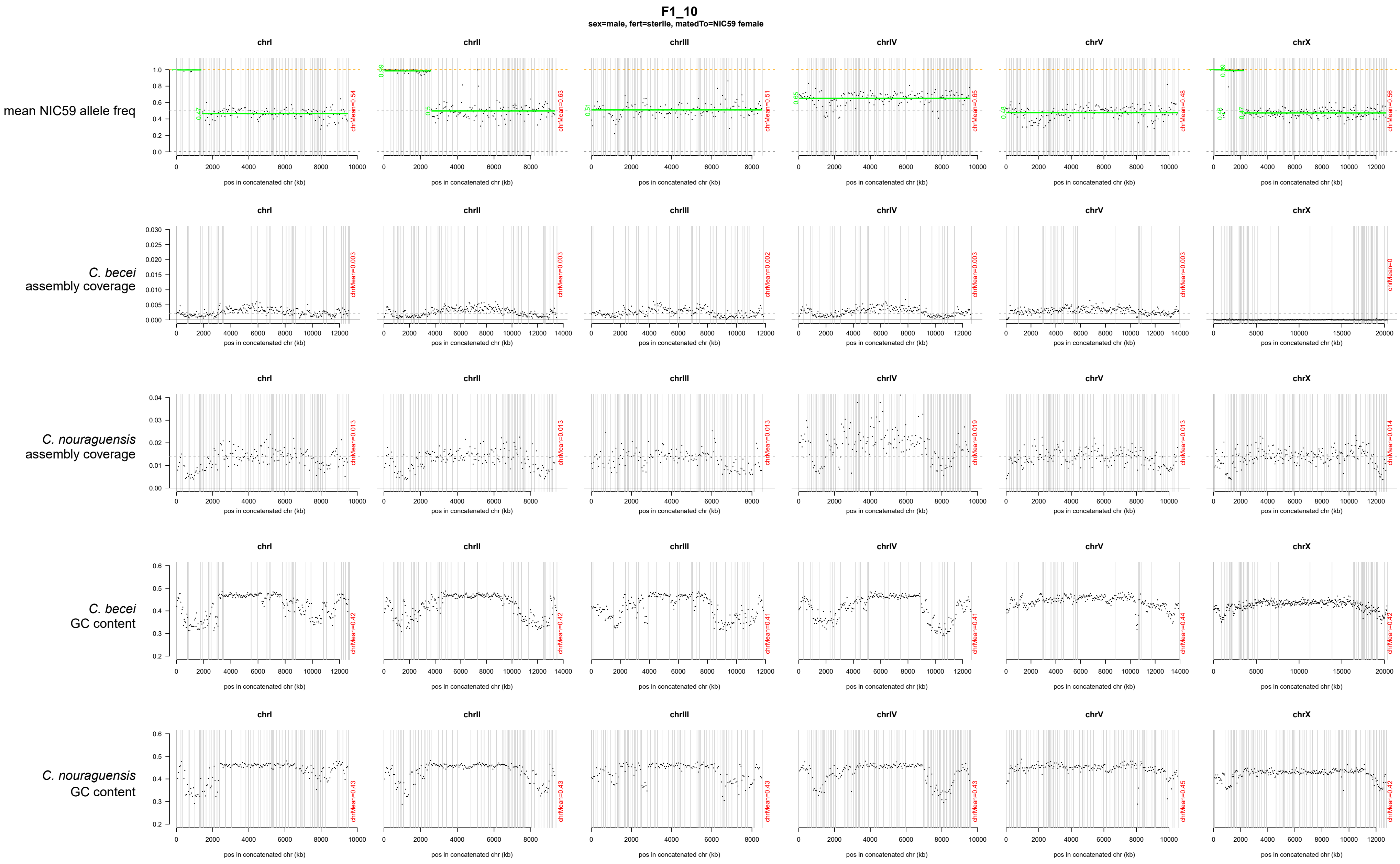

F1_10
sex=male, fert=sterile, matedTo=NIC59 female
mean NIC59 allele freq
C. becei assembly coverage
C. nouraguensis assembly coverage
C. becei GC content
C. nouraguensis GC content
chrI
chrII
chrIII
chrIV
chrV
chrX
pos in concatenated chr (kb)
chrMean=0.54
chrMean=0.63
chrMean=0.51
chrMean=0.65
chrMean=0.48
chrMean=0.56
chrMean=0.003
chrMean=0.003
chrMean=0.002
chrMean=0.003
chrMean=0.003
chrMean=0
chrMean=0.013
chrMean=0.013
chrMean=0.013
chrMean=0.019
chrMean=0.013
chrMean=0.014
chrMean=0.42
chrMean=0.42
chrMean=0.41
chrMean=0.41
chrMean=0.44
chrMean=0.42
chrMean=0.43
chrMean=0.43
chrMean=0.43
chrMean=0.43
chrMean=0.45
chrMean=0.42

F1_11

sex=female, fert=fertile, matedTo=NIC59 male

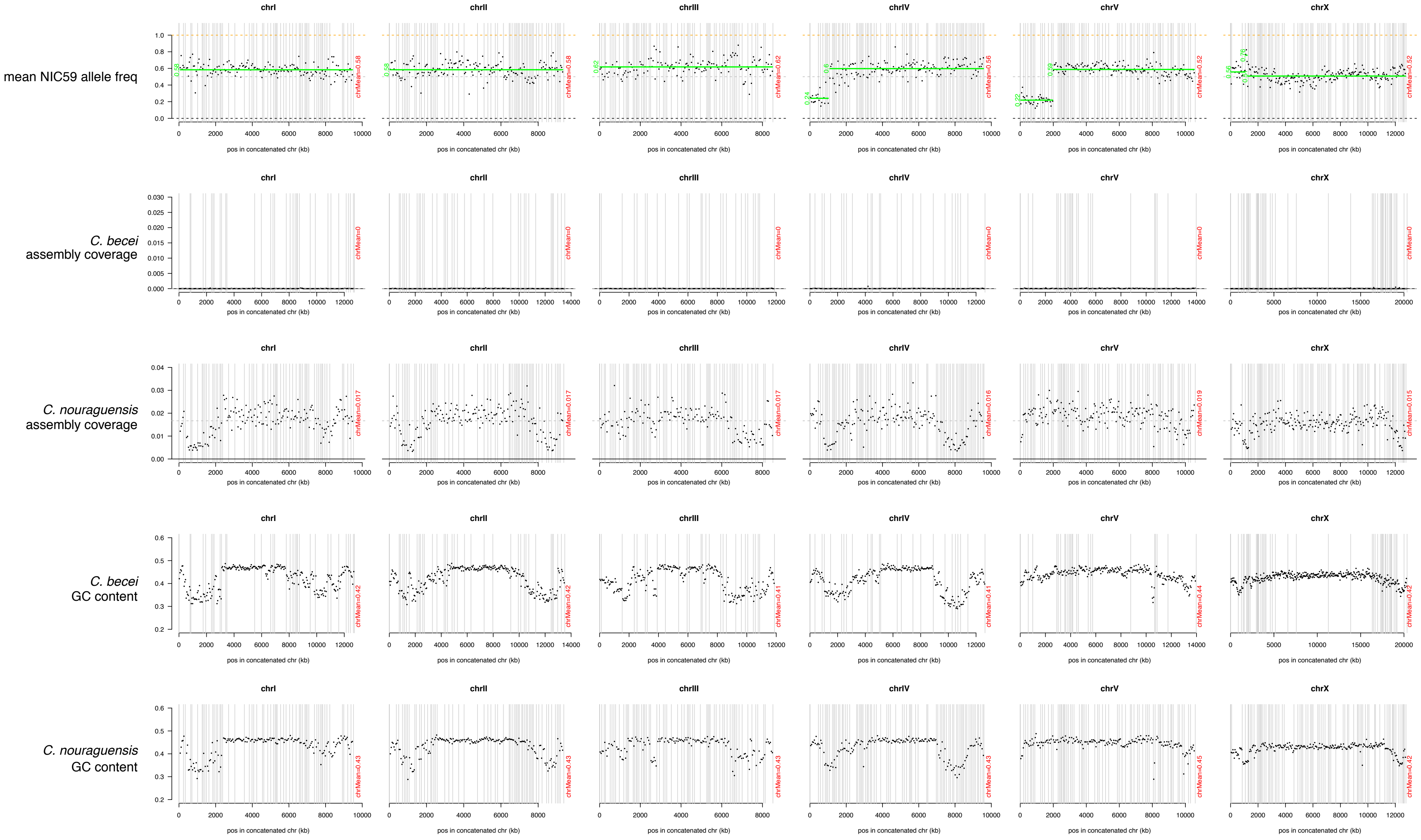

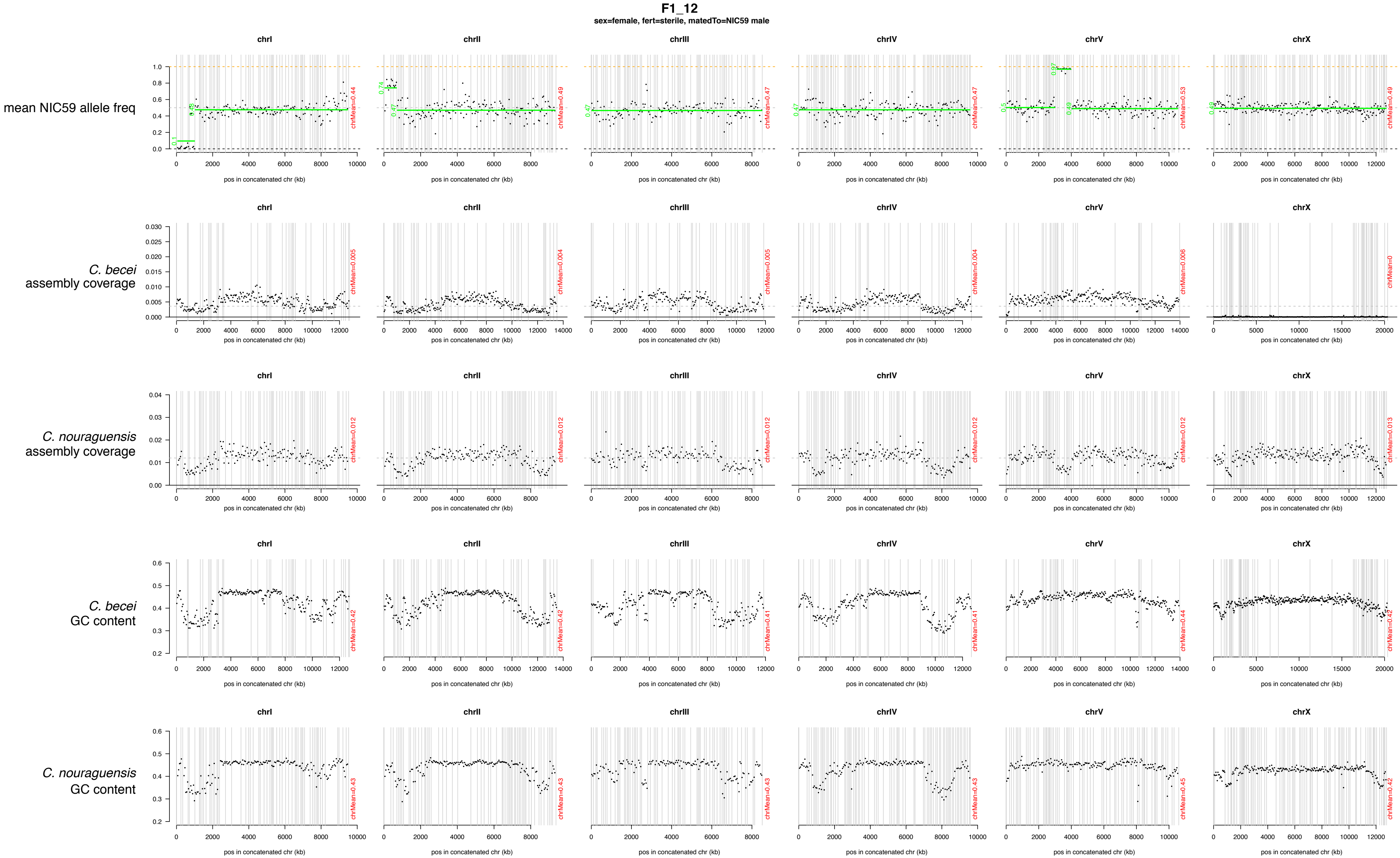
F1_12
sex=female, fert=sterile, matedTo=NIC59 male
chrI
chrII
chrIII
chrIV
chrV
chrX
mean NIC59 allele freq
C. becei assembly coverage
C. nouraguensis assembly coverage
C. becei GC content
C. nouraguensis GC content
pos in concatenated chr (kb)

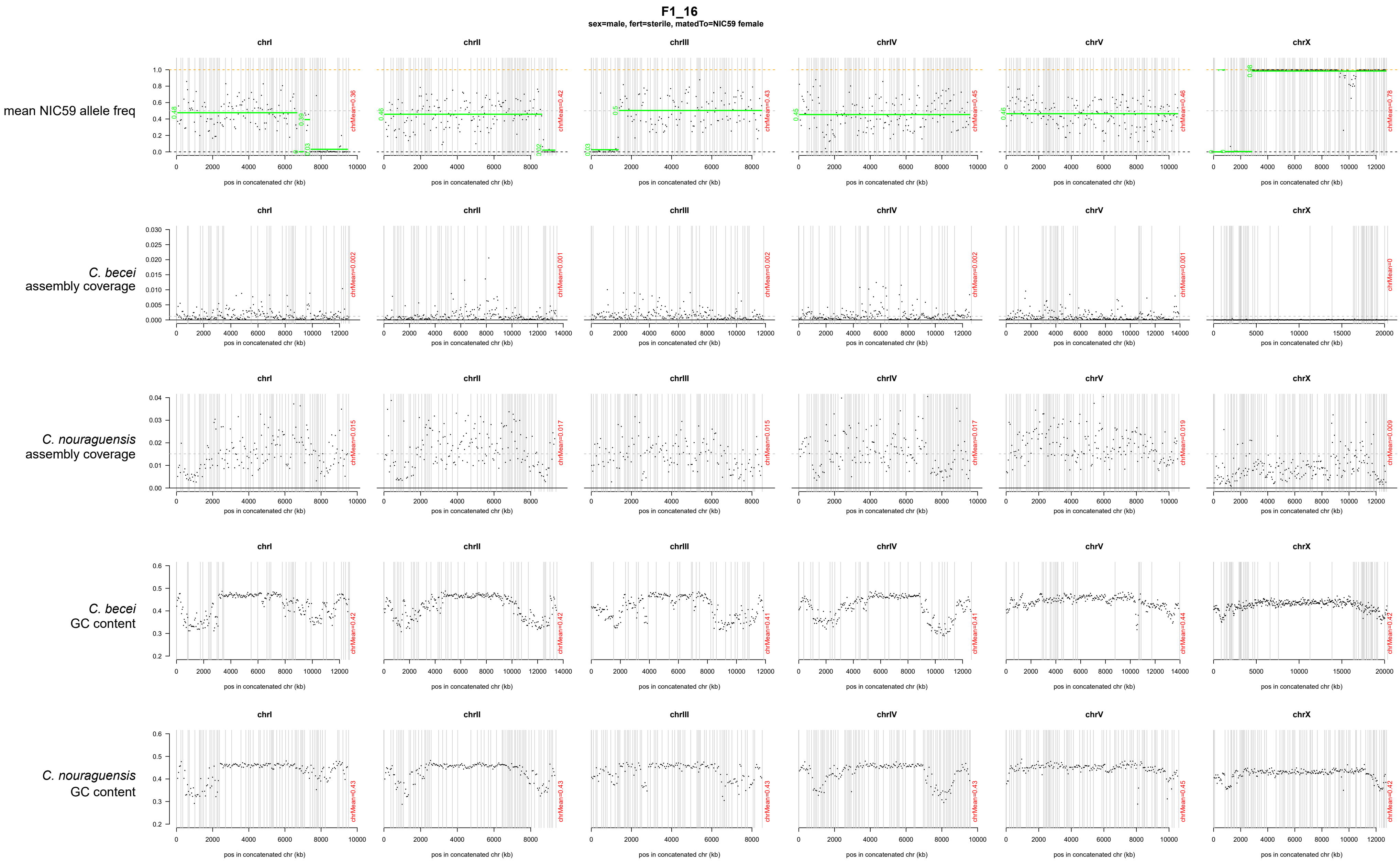
F1_16
sex=male, fert=sterile, matedTo=NIC59 female
mean NIC59 allele freq
C. becei assembly coverage
C. nouraguensis assembly coverage
C. becei GC content
C. nouraguensis GC content
chrI
chrII
chrIII
chrIV
chrV
chrX
pos in concatenated chr (kb)

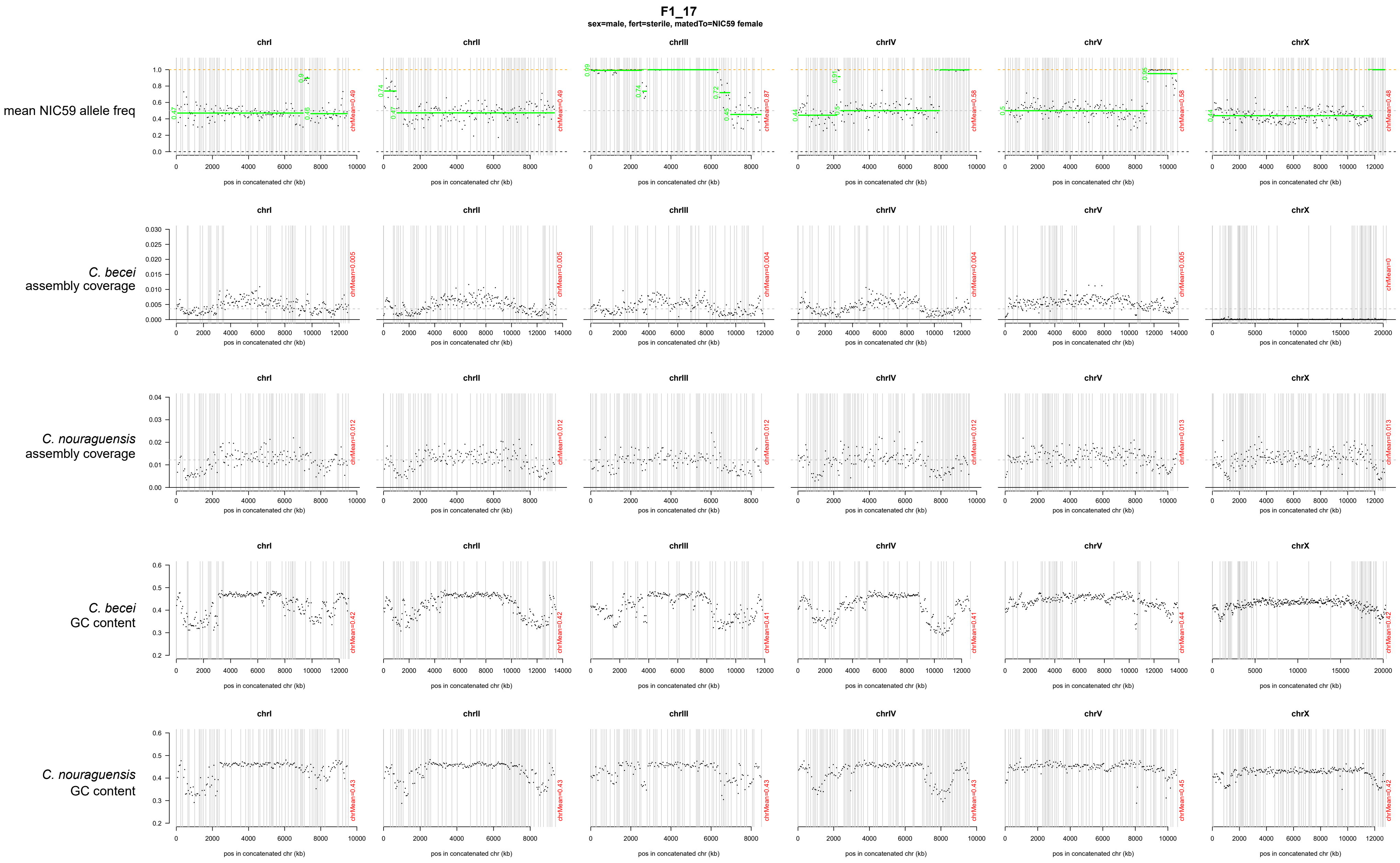
F1_17
sex=male, fert=sterile, matedTo=NIC59 female
chrI
chrII
chrIII
chrIV
chrV
chrX
mean NIC59 allele freq
C. becei assembly coverage
C. nouraguensis assembly coverage
C. becei GC content
C. nouraguensis GC content
pos in concatenated chr (kb)
chrMean=0.49
chrMean=0.49
chrMean=0.87
chrMean=0.58
chrMean=0.58
chrMean=0.48
chrMean=0.005
chrMean=0.005
chrMean=0.004
chrMean=0.004
chrMean=0.005
chrMean=0
chrMean=0.012
chrMean=0.012
chrMean=0.012
chrMean=0.012
chrMean=0.013
chrMean=0.013
chrMean=0.42
chrMean=0.42
chrMean=0.41
chrMean=0.41
chrMean=0.44
chrMean=0.42
chrMean=0.43
chrMean=0.43
chrMean=0.43
chrMean=0.43
chrMean=0.45
chrMean=0.42

F1_18

sex=female, fert=sterile, matedTo=JU1825 male

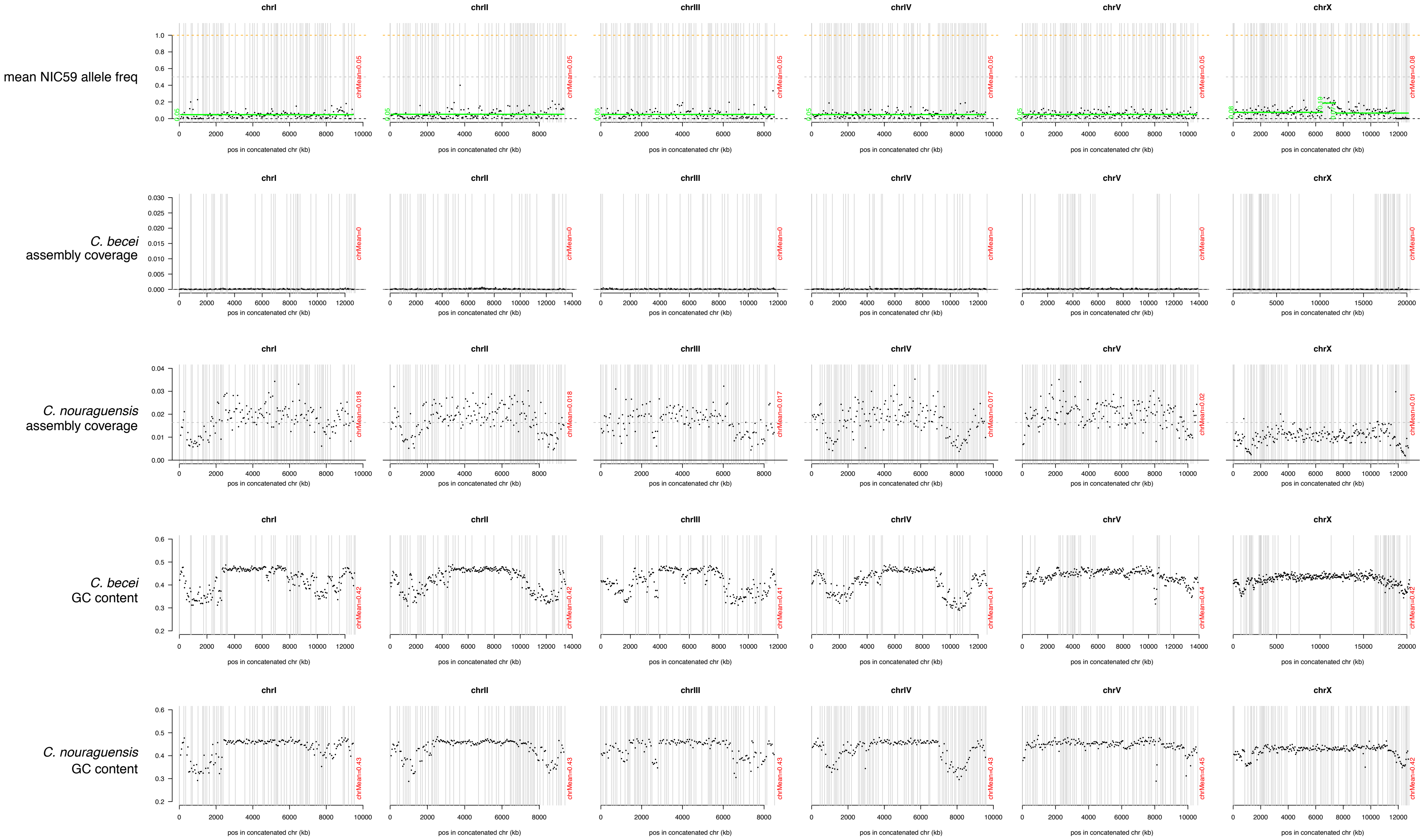

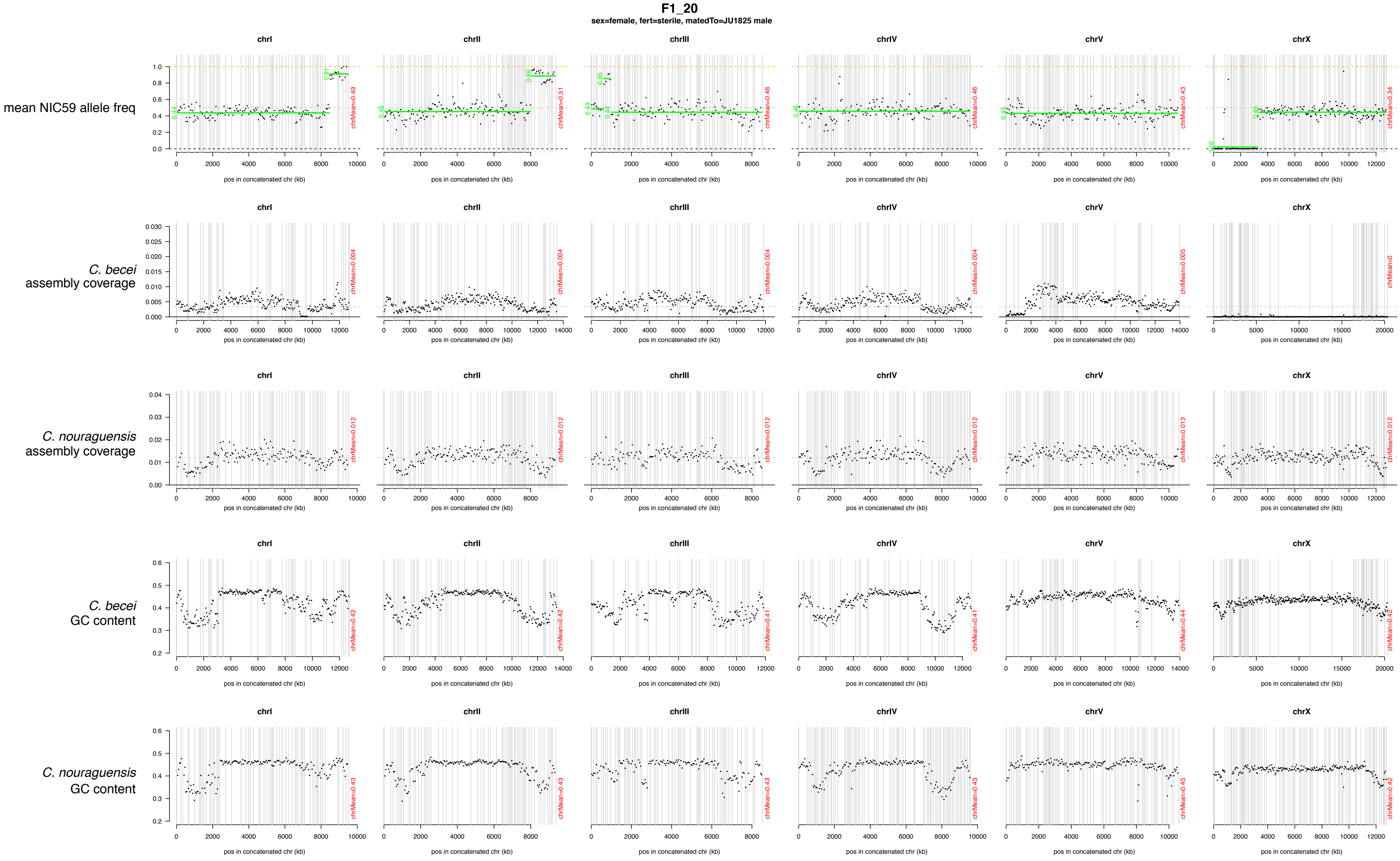
F1_20
sex=female, fert=sterile, matedTo=JU1825 male
mean NIC59 allele freq
C. becei assembly coverage
C. nouraguensis assembly coverage
C. becei GC content
C. nouraguensis GC content
chrI
chrII
chrIII
chrIV
chrV
chrX
pos in concatenated chr (kb)

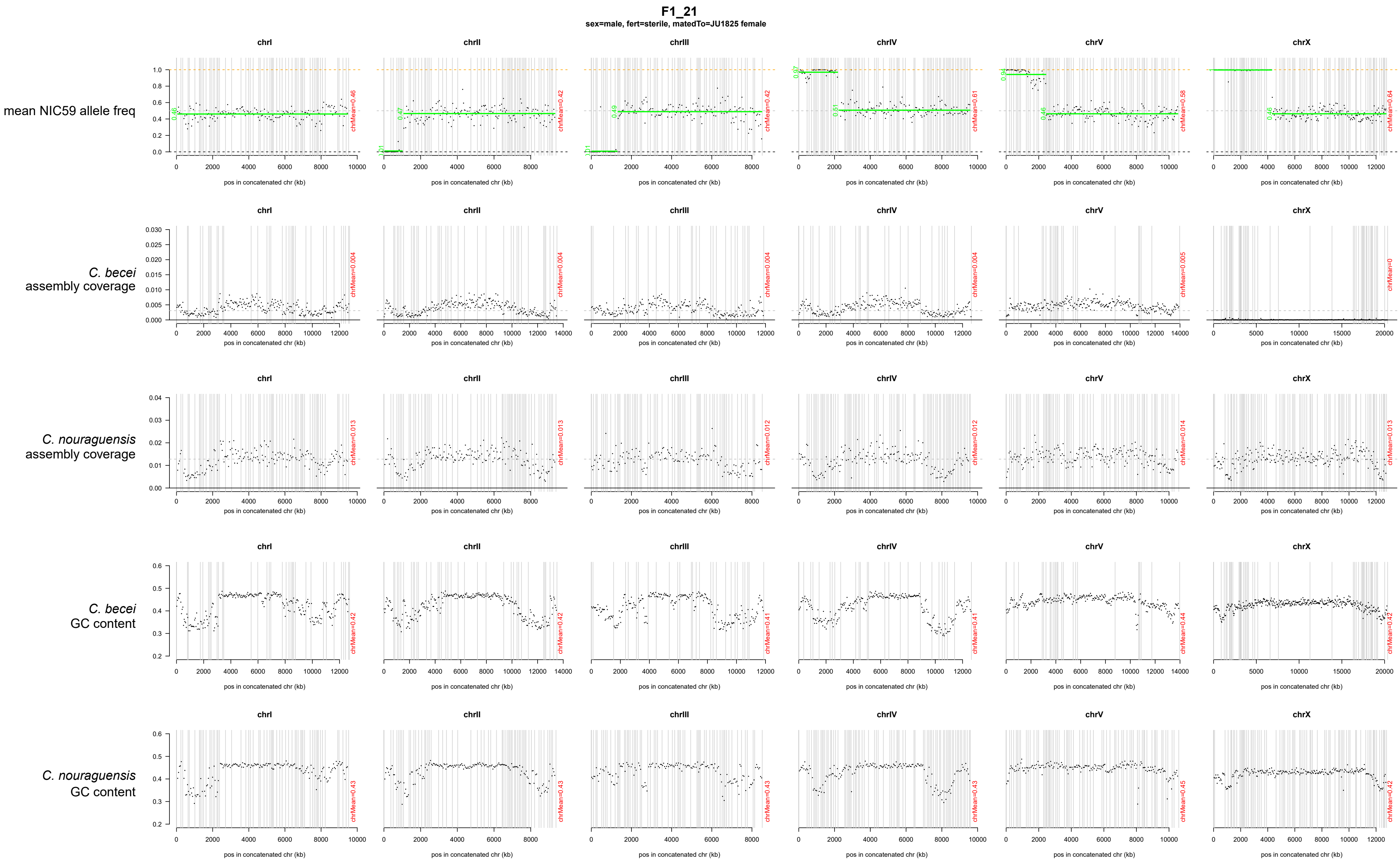
F1_21
sex=male, fert=sterile, matedTo=JU1825 female
mean NIC59 allele freq
C. becei assembly coverage
C. nouraguensis assembly coverage
C. becei GC content
C. nouraguensis GC content
chrI
chrII
chrIII
chrIV
chrV
chrX
pos in concatenated chr (kb)

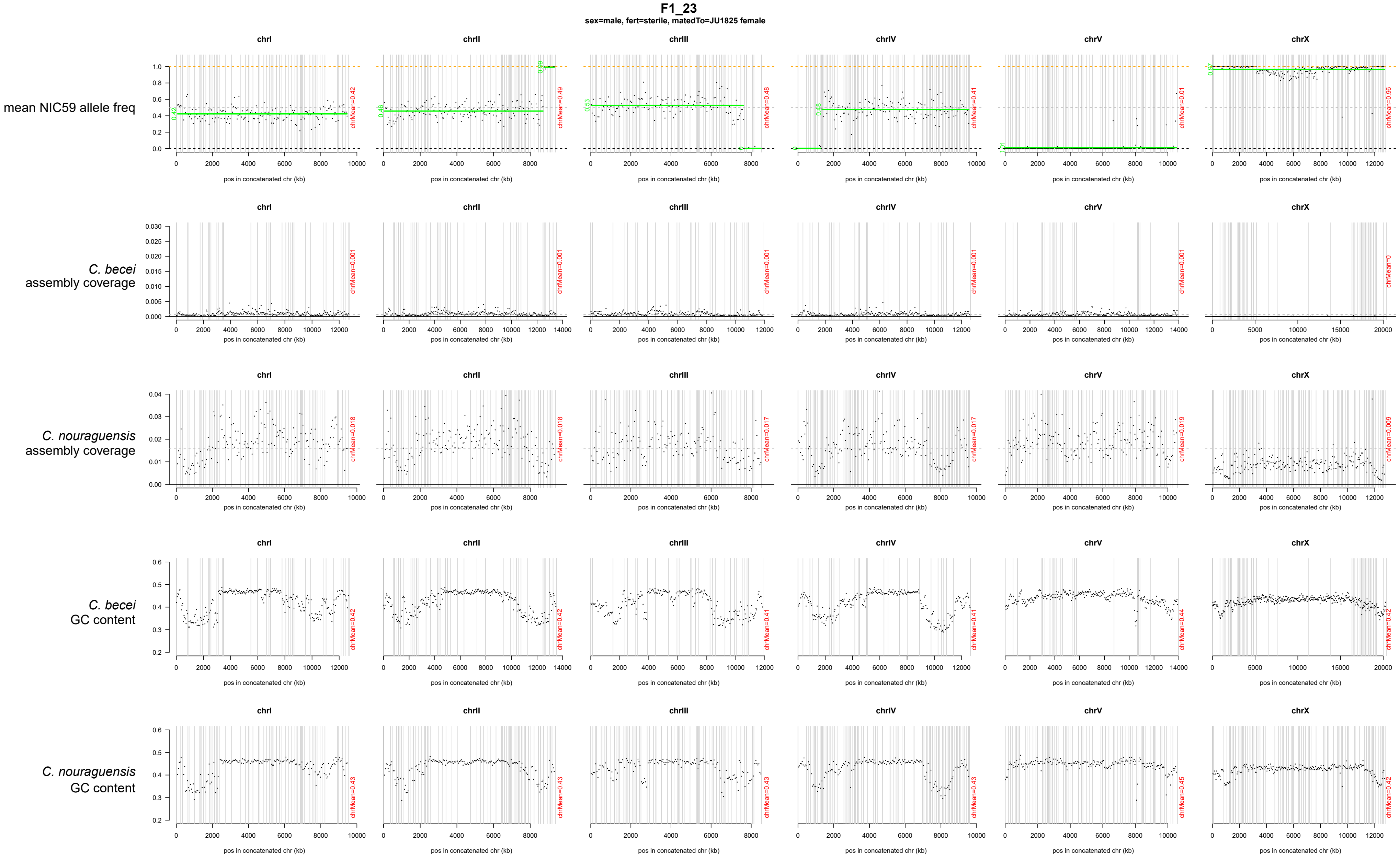
F1_23
sex=male, fert=sterile, matedTo=JU1825 female
chrI
chrII
chrIII
chrIV
chrV
chrX
mean NIC59 allele freq
C. becei assembly coverage
C. nouraguensis assembly coverage
C. becei GC content
C. nouraguensis GC content
pos in concatenated chr (kb)

F1_25

sex=female, fert=fertile, matedTo=JU1825 male

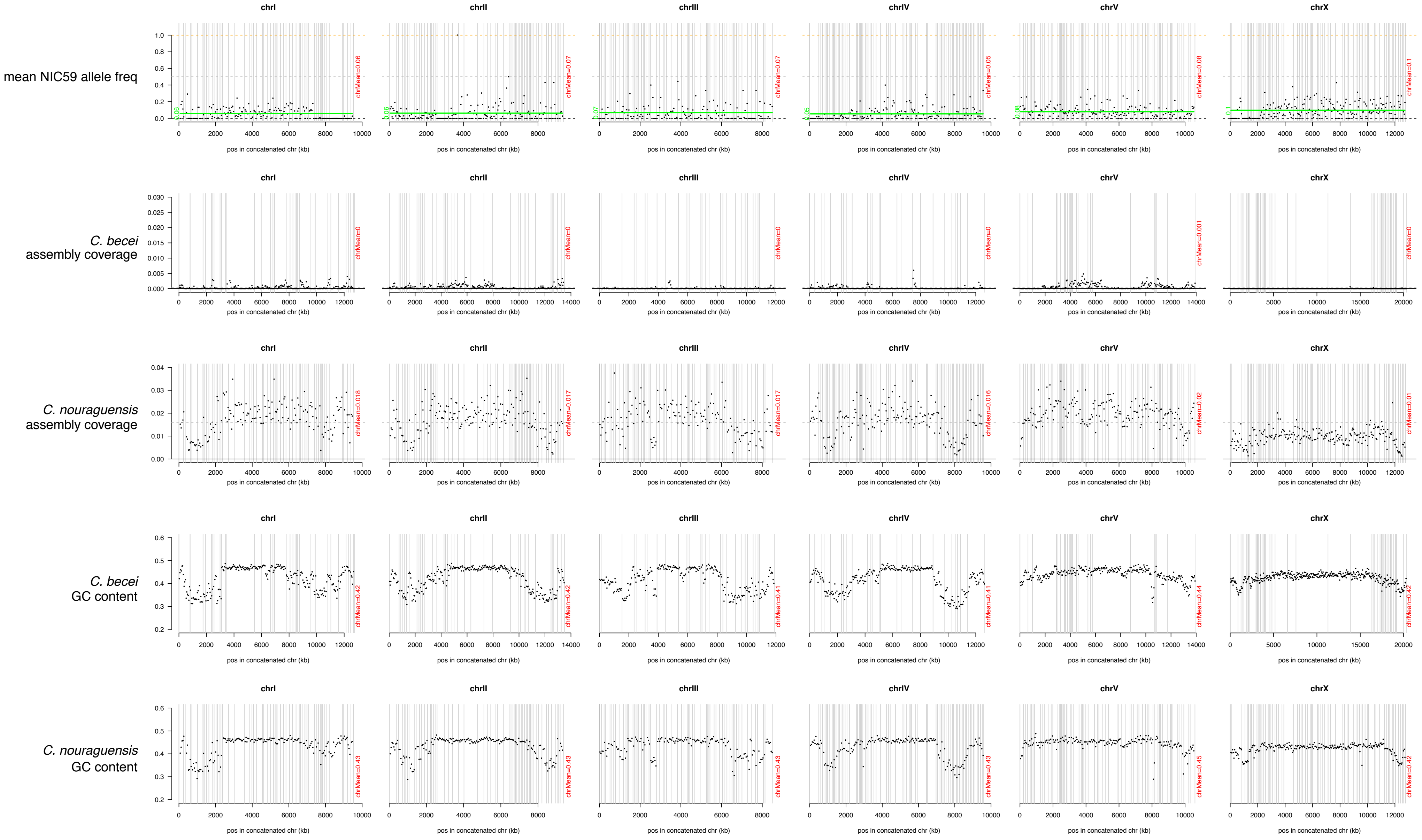

F1_26

sex=female, fert=sterile, matedTo=JU1825 male

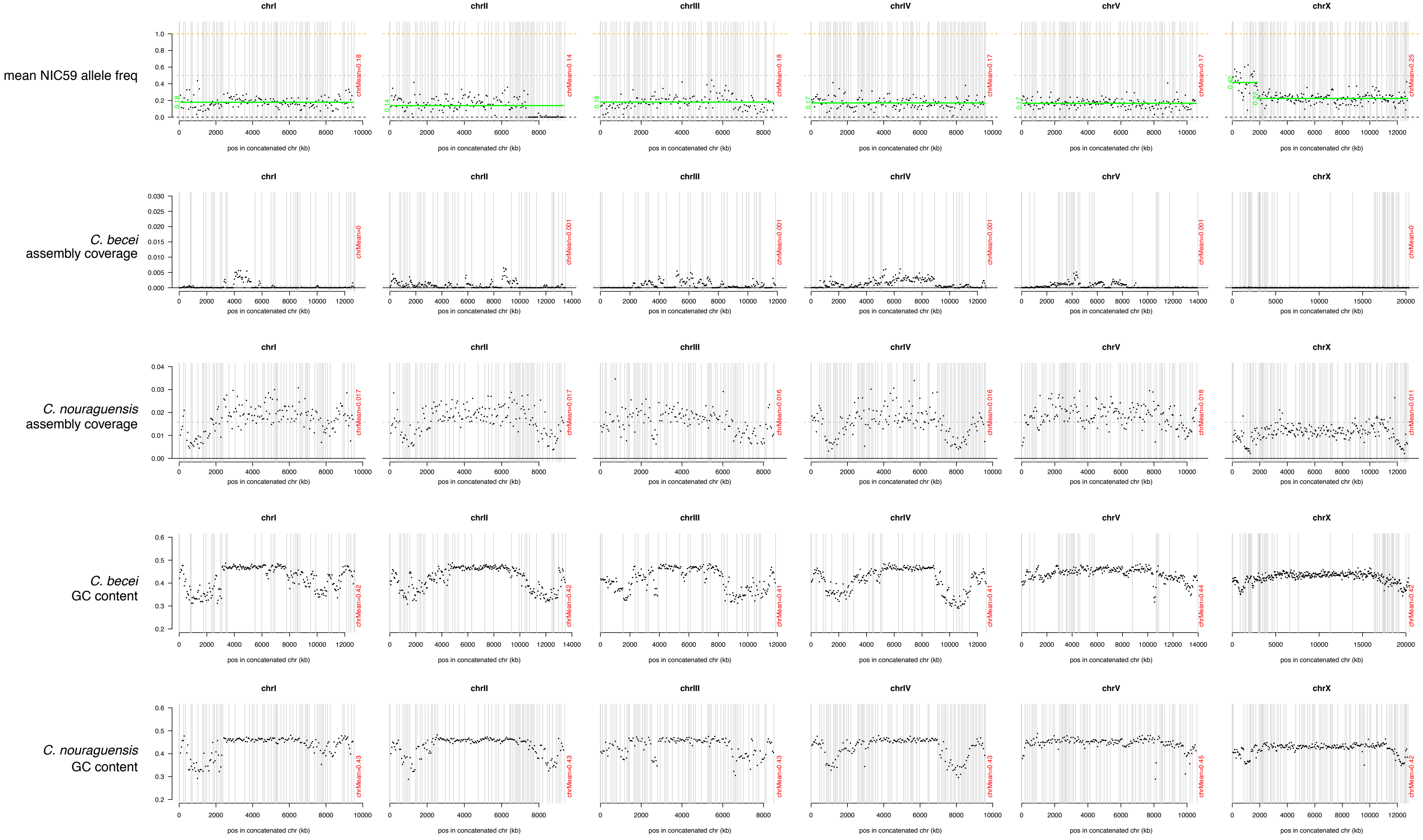

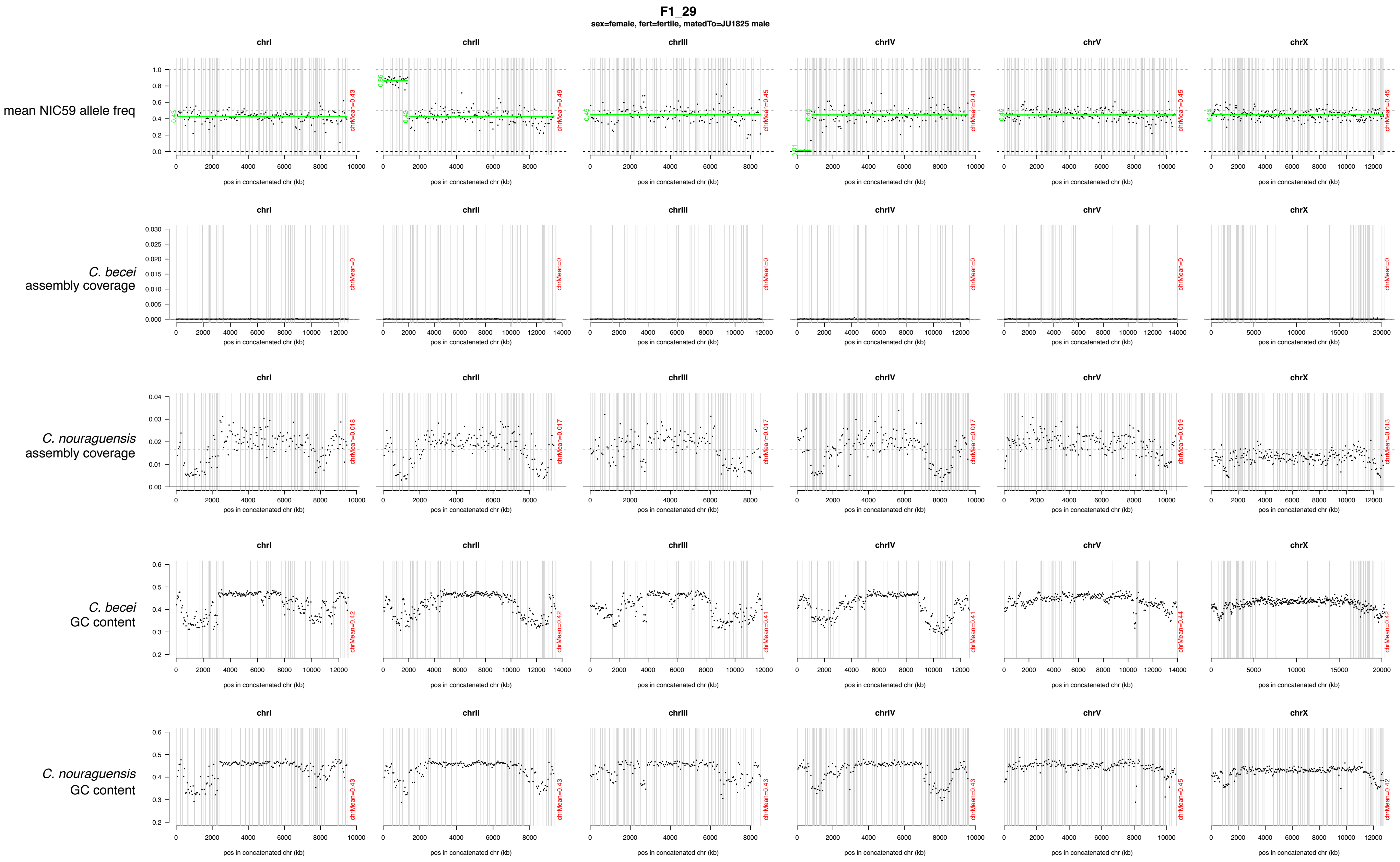
F1_29
sex=female, fert=fertile, matedTo=JU1825 male
mean NIC59 allele freq
C. becei assembly coverage
C. nouraguensis assembly coverage
C. becei GC content
C. nouraguensis GC content
chrI
chrII
chrIII
chrIV
chrV
chrX
pos in concatenated chr (kb)

F1_39

sex=female, fert=fertile, matedTo=JU1825 male

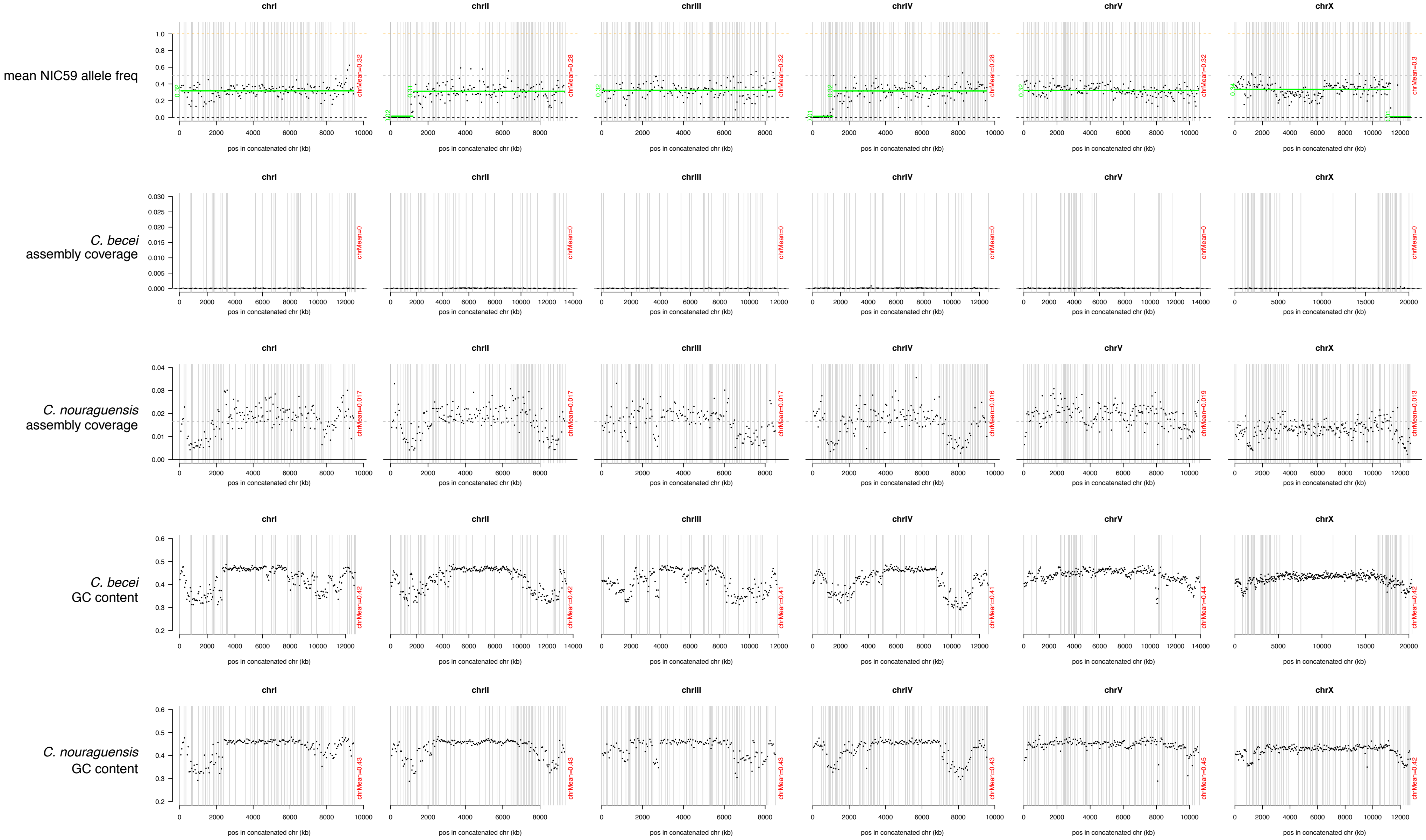

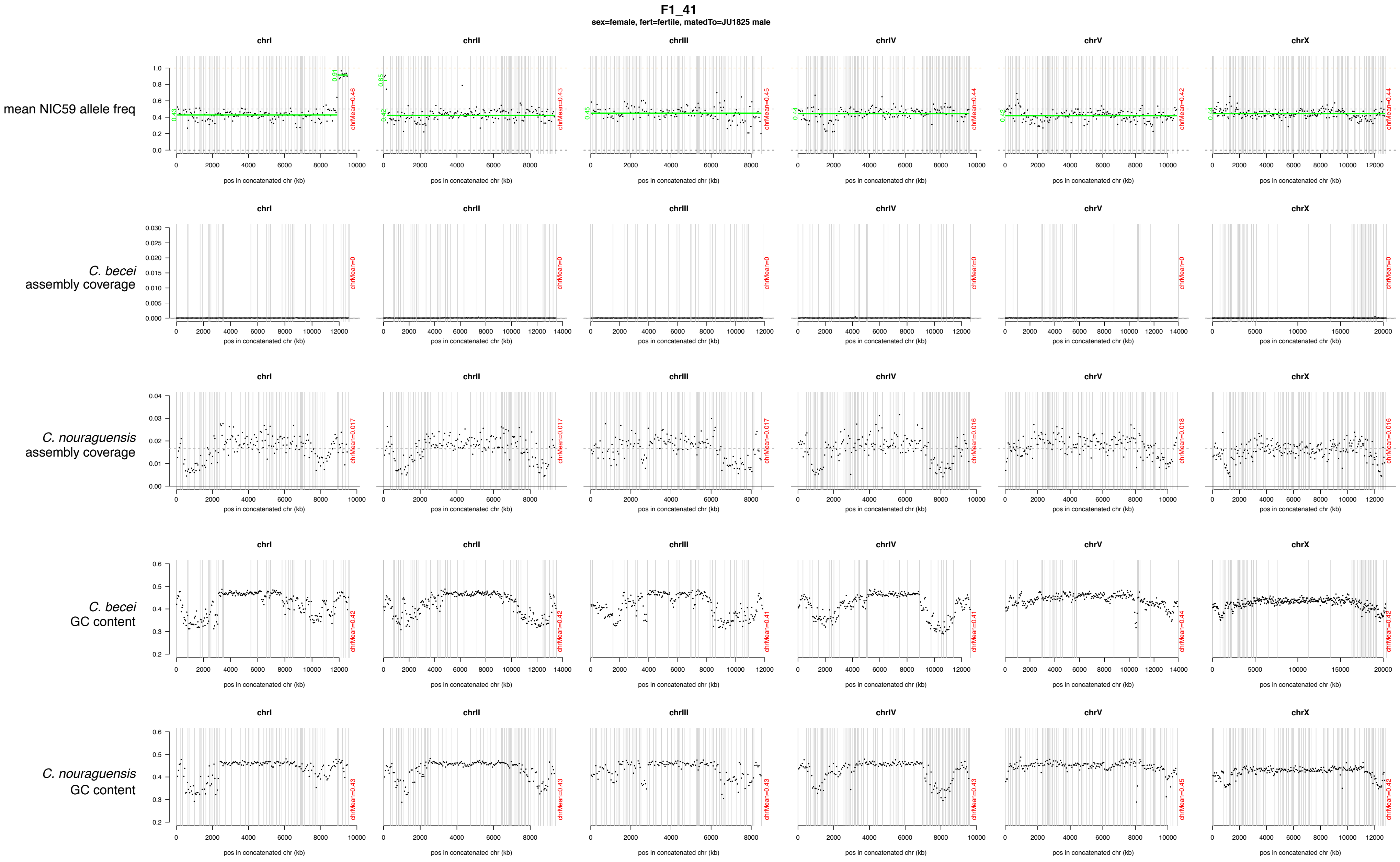
F1_41
sex=female, fert=fertile, matedTo=JU1825 male
mean NIC59 allele freq
C. becei assembly coverage
C. nouraguensis assembly coverage
C. becei GC content
C. nouraguensis GC content
chrI
chrII
chrIII
chrIV
chrV
chrX
pos in concatenated chr (kb)
chrMean=0.46
chrMean=0.43
chrMean=0.45
chrMean=0.44
chrMean=0.42
chrMean=0.44
chrMean=0
chrMean=0.017
chrMean=0.017
chrMean=0.017
chrMean=0.016
chrMean=0.018
chrMean=0.016
chrMean=0.42
chrMean=0.42
chrMean=0.41
chrMean=0.41
chrMean=0.44
chrMean=0.42
chrMean=0.43
chrMean=0.43
chrMean=0.43
chrMean=0.43
chrMean=0.45
chrMean=0.42

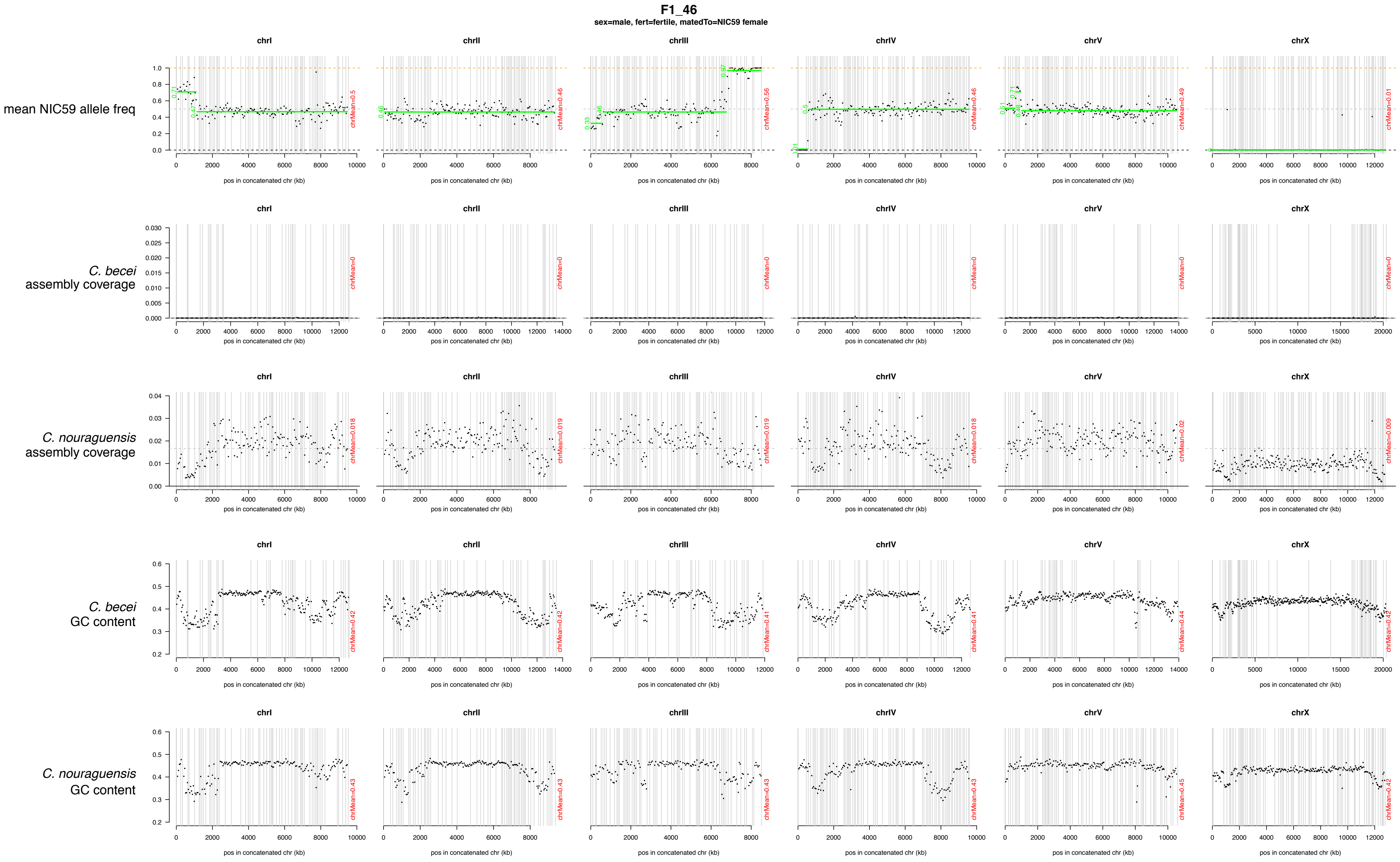
F1_46
sex=male, fert=fertile, matedTo=NIC59 female
mean NIC59 allele freq
C. becei assembly coverage
C. nouraguensis assembly coverage
C. becei GC content
C. nouraguensis GC content
chrI
chrII
chrIII
chrIV
chrV
chrX
pos in concatenated chr (kb)
chrMean=0.5
chrMean=0.46
chrMean=0.56
chrMean=0.46
chrMean=0.49
chrMean=0.01
chrMean=0
chrMean=0.018
chrMean=0.019
chrMean=0.019
chrMean=0.018
chrMean=0.02
chrMean=0.009
chrMean=0.42
chrMean=0.42
chrMean=0.41
chrMean=0.41
chrMean=0.44
chrMean=0.42
chrMean=0.43
chrMean=0.43
chrMean=0.43
chrMean=0.43
chrMean=0.45
chrMean=0.42

F1_48

sex=male, fert=fertile, matedTo=NIC59 female

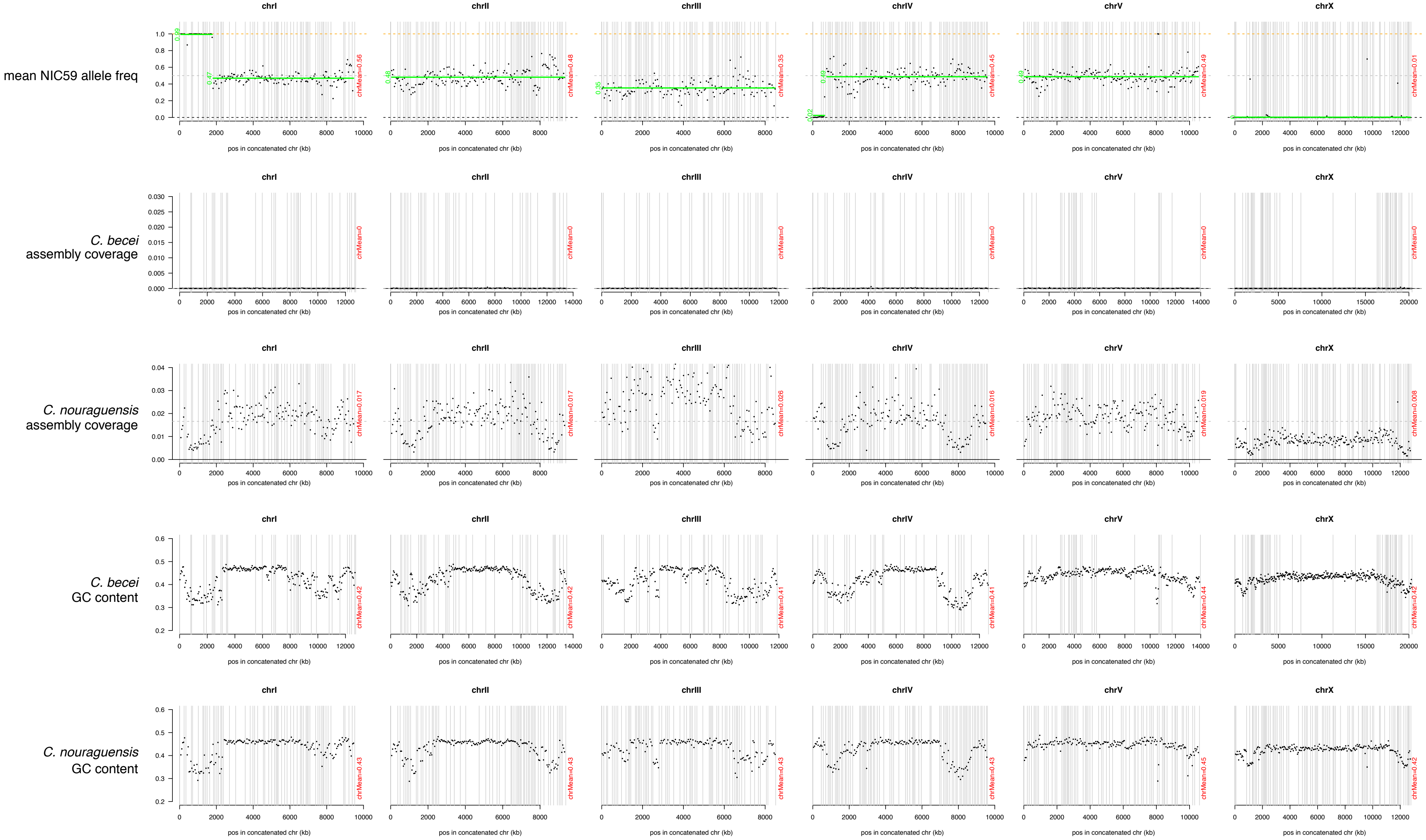

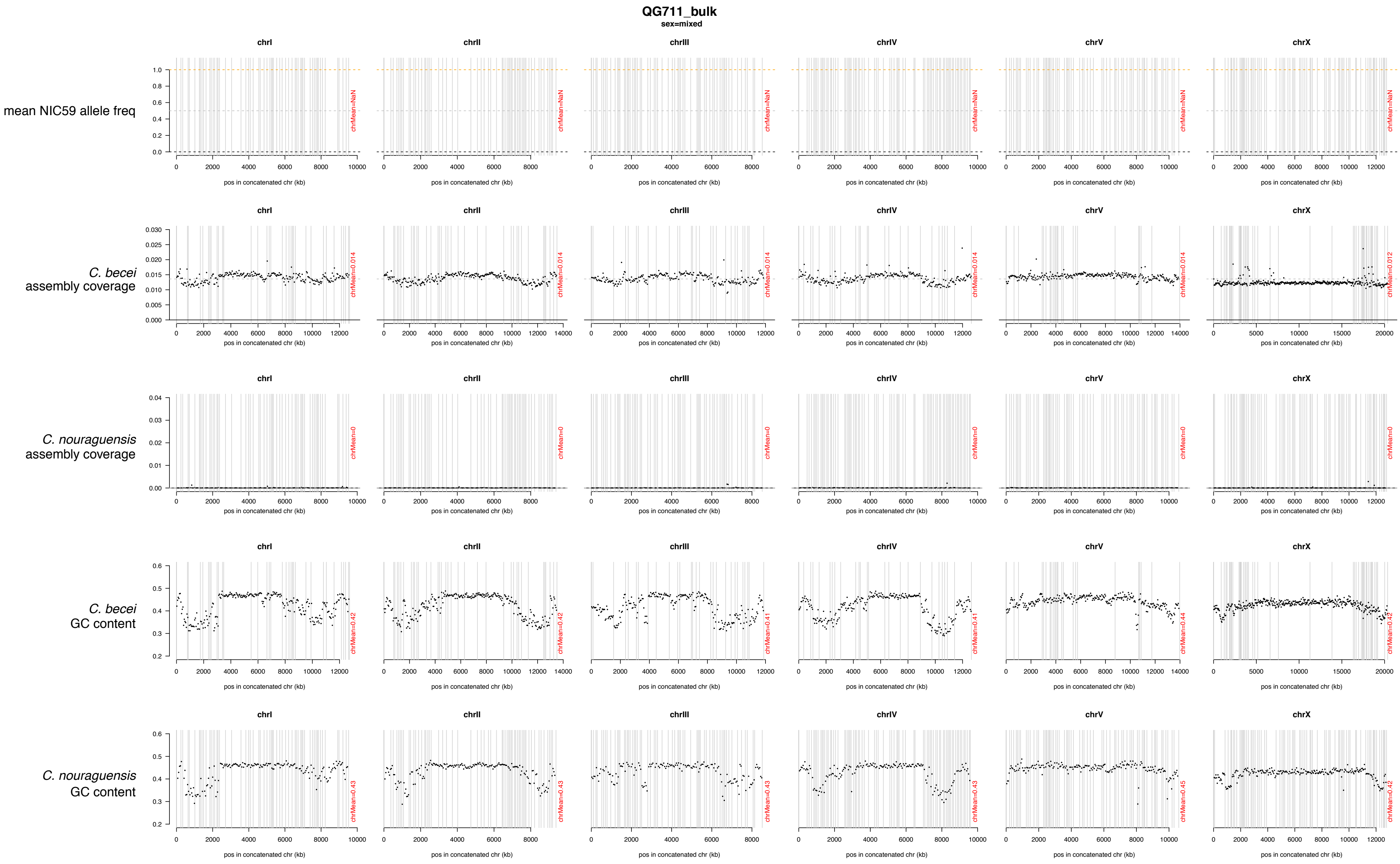
QG711_bulk
sex=mixed
mean NIC59 allele freq
C. becei
assembly coverage
C. nouraguensis
assembly coverage
C. becei
GC content
C. nouraguensis
GC content
chrI
chrII
chrIII
chrIV
chrV
chrX
pos in concatenated chr (kb)
chrMean=NaN
chrMean=0.014
chrMean=0.012
chrMean=0
chrMean=0.42
chrMean=0.41
chrMean=0.44
chrMean=0.43
chrMean=0.45

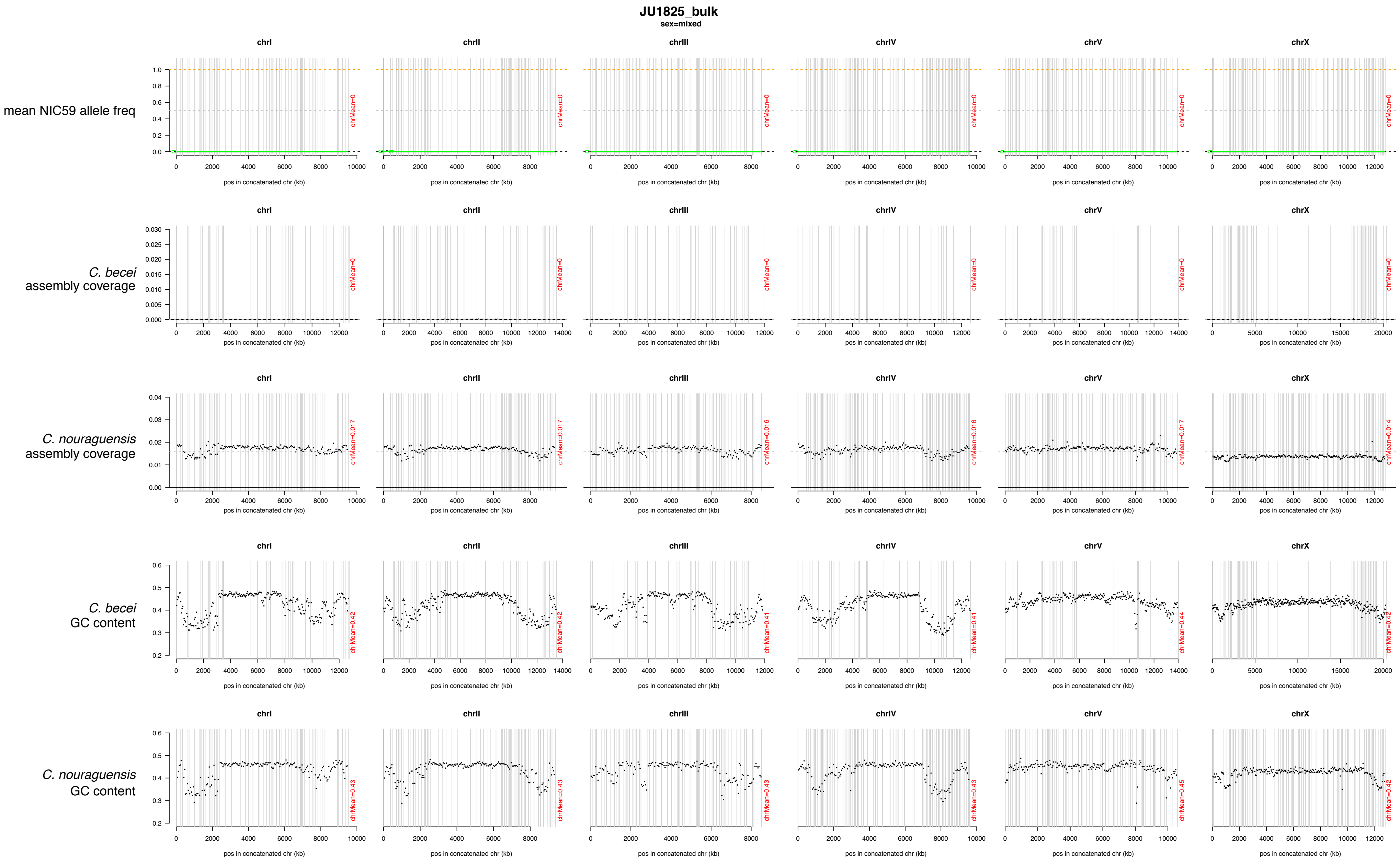
JU1825_bulk
sex=mixed
chrI
chrII
chrIII
chrIV
chrV
chrX
mean NIC59 allele freq
C. becei assembly coverage
C. nouraguensis assembly coverage
C. becei GC content
C. nouraguensis GC content
pos in concatenated chr (kb)
chrMean=0
chrMean=0.017
chrMean=0.016
chrMean=0.014
chrMean=0.42
chrMean=0.41
chrMean=0.44
chrMean=0.43
chrMean=0.45

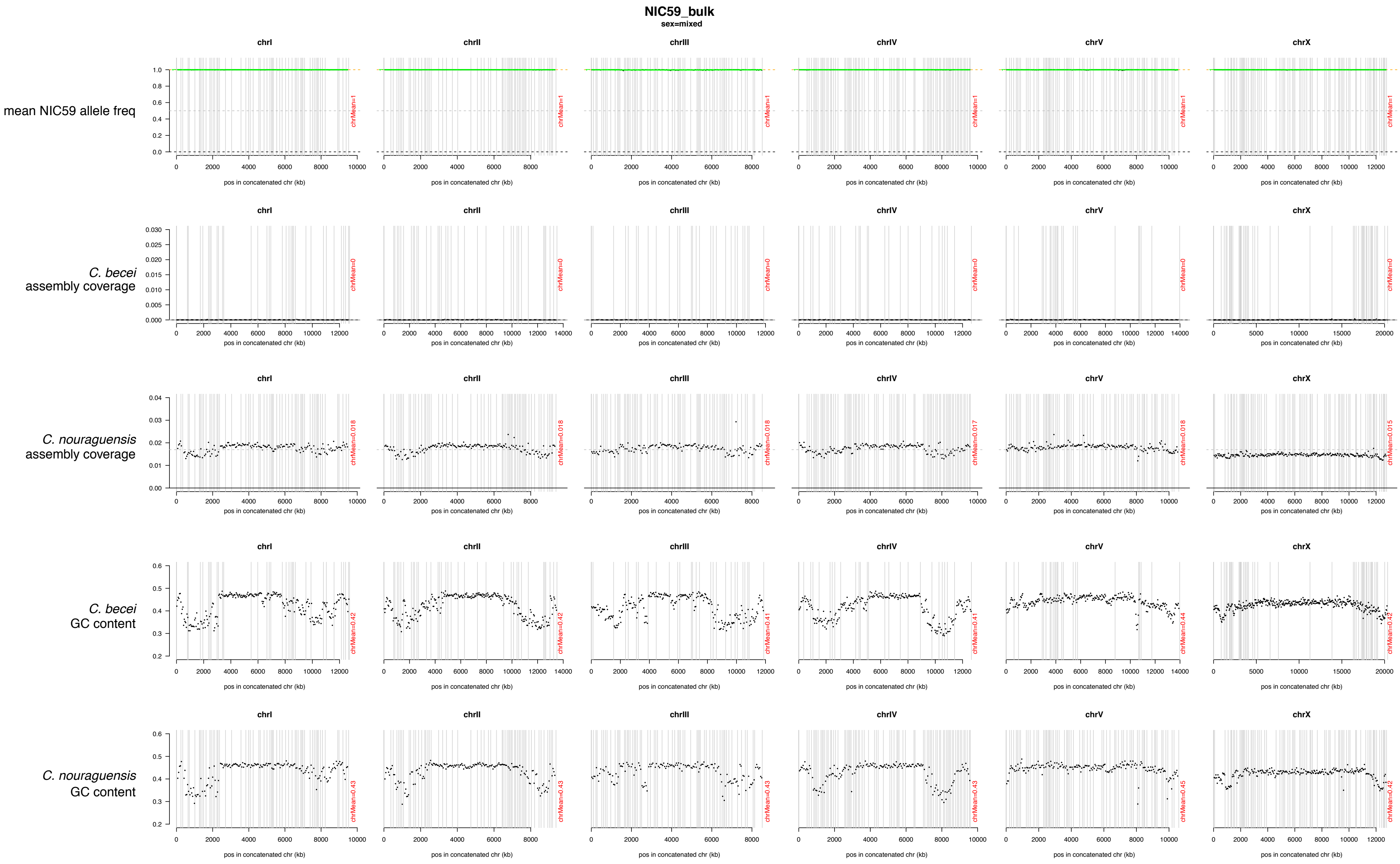

NIC59_bulk
sex=mixed
mean NIC59 allele freq
C. becei assembly coverage
C. nouraguensis assembly coverage
C. becei GC content
C. nouraguensis GC content
chrI
chrII
chrIII
chrIV
chrV
chrX
pos in concatenated chr (kb)

NIC59plusJU1825plusQG711

sex=female

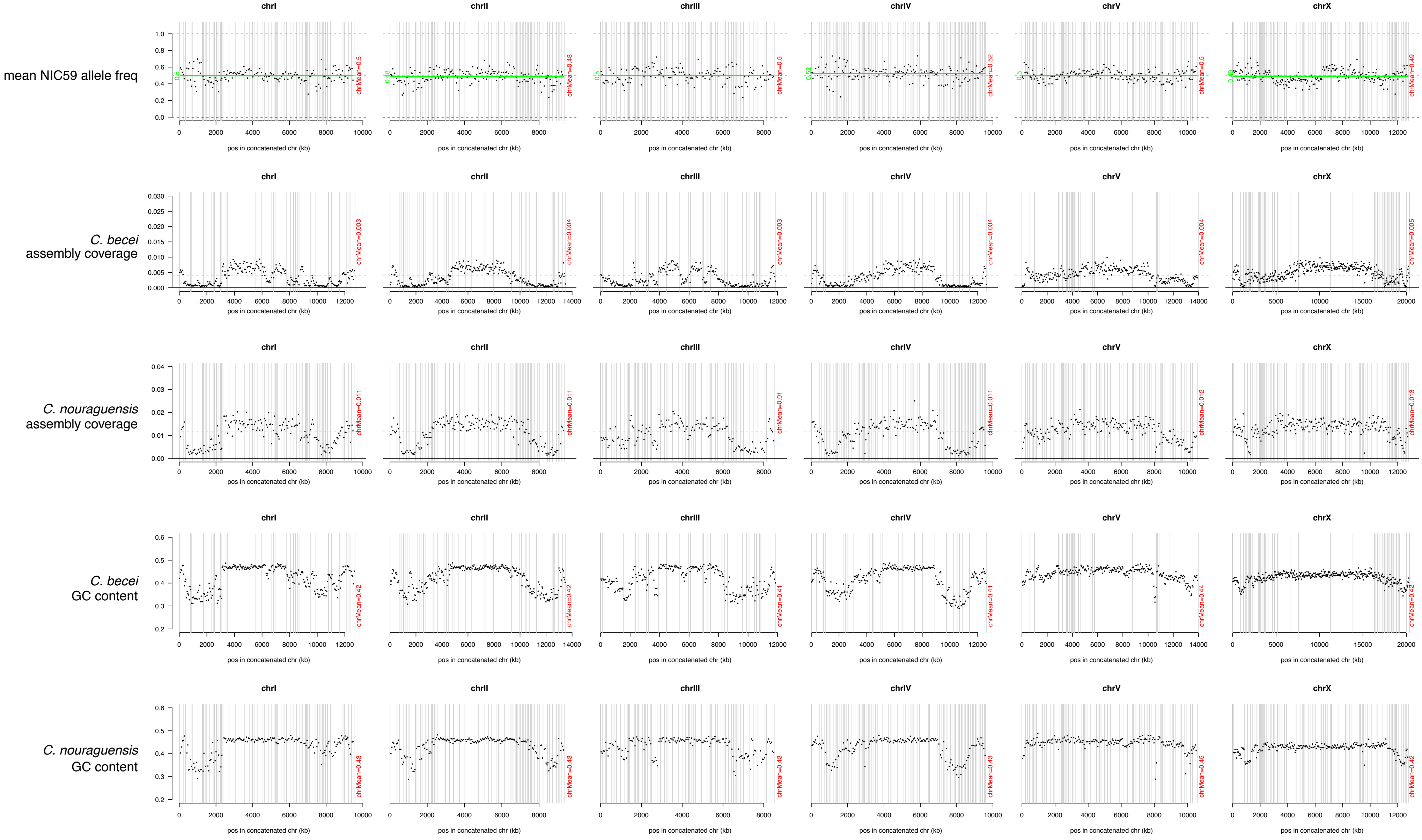

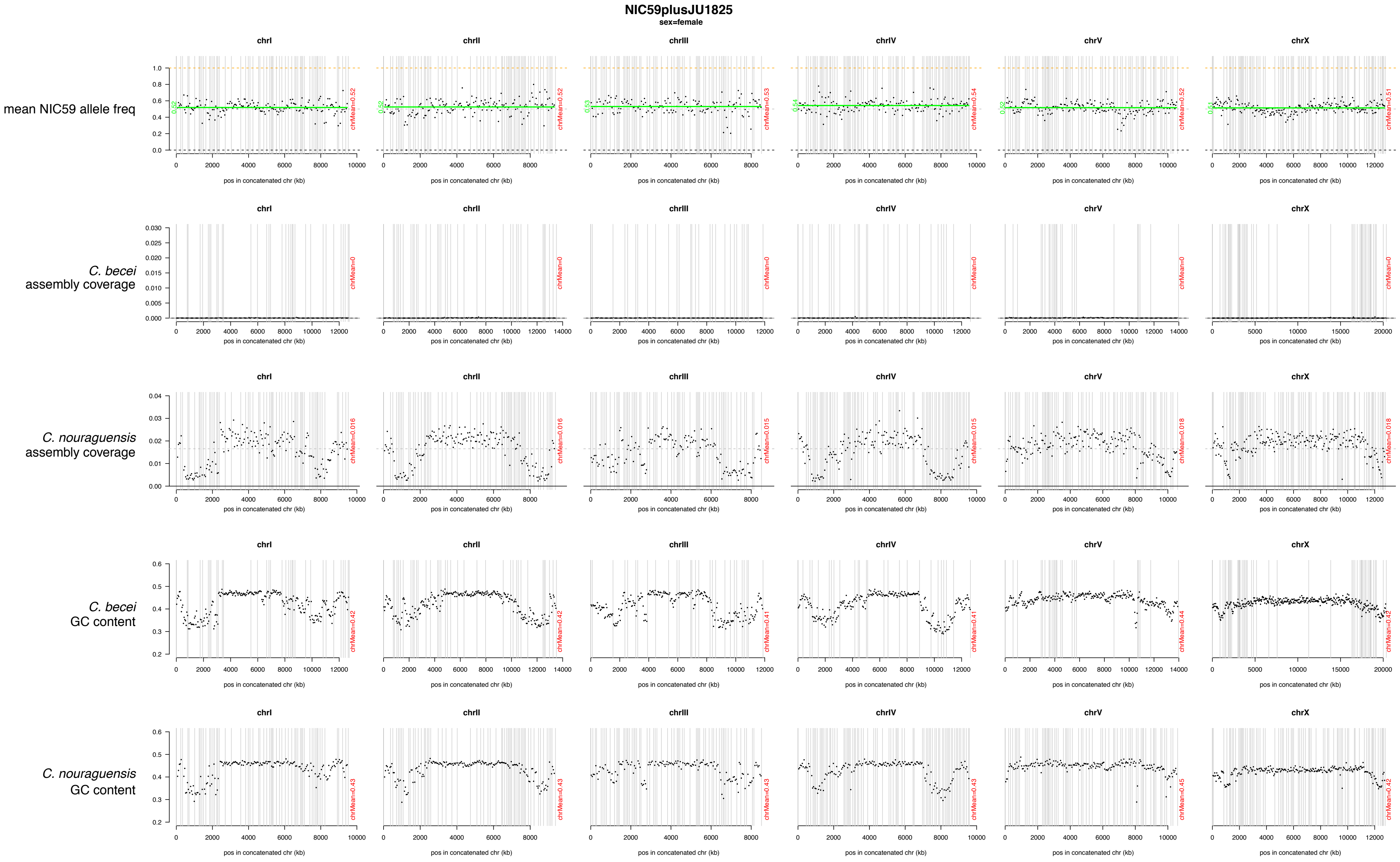
NIC59plusJU1825
sex=female
chrI
chrII
chrIII
chrIV
chrV
chrX
mean NIC59 allele freq
C. becei
assembly coverage
C. nouraguensis
assembly coverage
C. becei
GC content
C. nouraguensis
GC content
pos in concatenated chr (kb)
chrMean=0.52
chrMean=0.52
chrMean=0.53
chrMean=0.54
chrMean=0.52
chrMean=0.51
chrMean=0
chrMean=0.016
chrMean=0.016
chrMean=0.015
chrMean=0.015
chrMean=0.018
chrMean=0.018
chrMean=0.42
chrMean=0.42
chrMean=0.41
chrMean=0.41
chrMean=0.44
chrMean=0.42
chrMean=0.43
chrMean=0.43
chrMean=0.43
chrMean=0.43
chrMean=0.45
chrMean=0.42

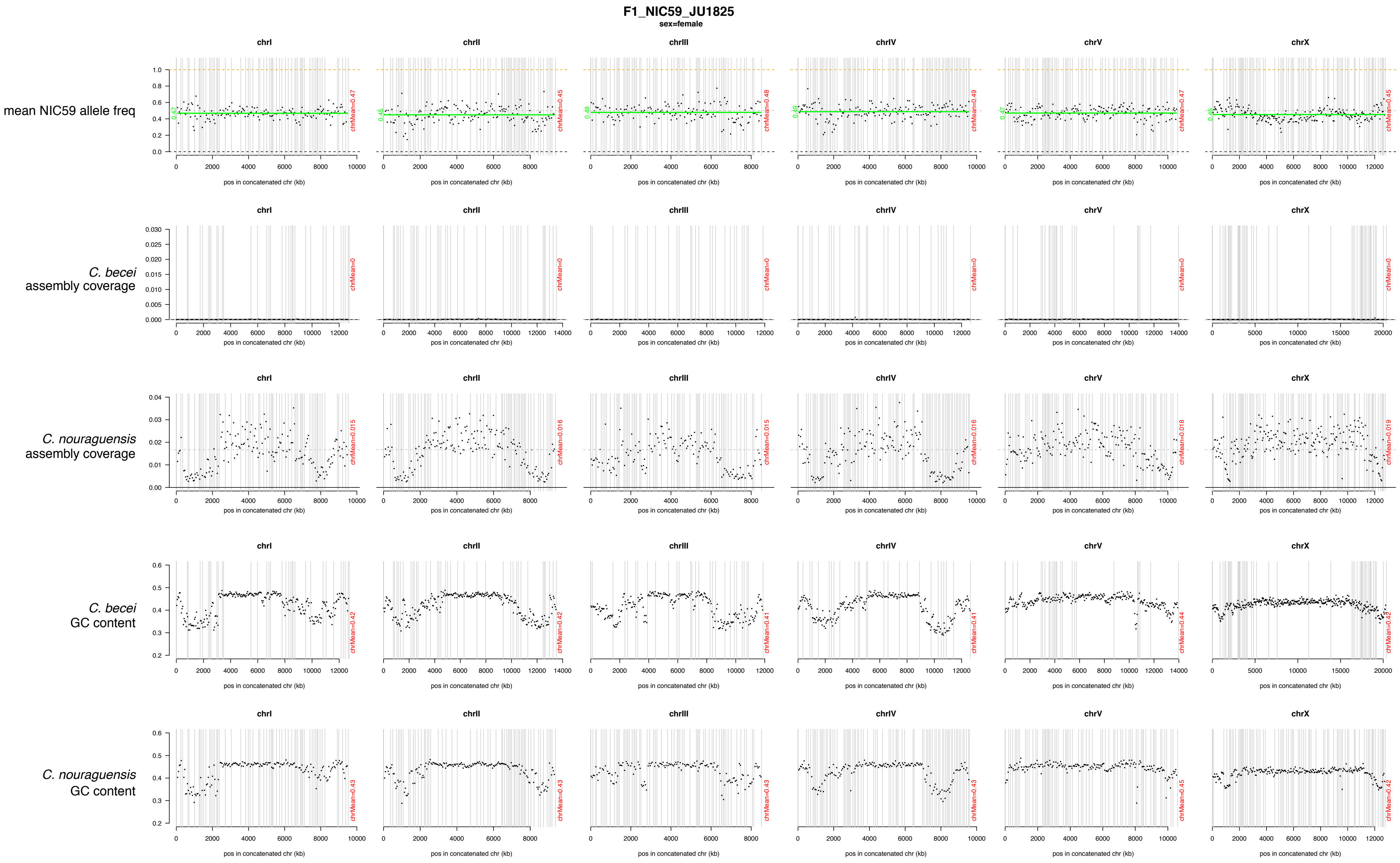
F1_NIC59_JU1825
sex=female
chrI
chrII
chrIII
chrIV
chrV
chrX
mean NIC59 allele freq
C. becei assembly coverage
C. nouraguensis assembly coverage
C. becei GC content
C. nouraguensis GC content
pos in concatenated chr (kb)
