# Supplementary material for: Hybridization promotes asexual reproduction in Caenorhabditis nematodes
Source: PLoS Genet. 2019 Dec 16;15(12):e1008520. doi: 10.1371/journal.pgen.1008520 (PMC6946170; doi:10.1371/journal.pgen.1008520)
Supplement: S8 Fig — (A) Fertilization with UV-irradiated sperm does not significantly increase diploid maternal inheritance in C. nouraguensis intraspecies crosses. Heterozygous C. nouraguensis (NIC59/JU1825) females were mated to UV-irradiated C. nouraguensis (NIC59) males. Dead embryos produced by this cross were PCR genotyped at a single autosomal locus (oPL78+79). The gel shows the genotypes of a fraction of the dead embryos assayed. Most embryos had either a JU1825 (11/31) or NIC59 genotype (17/31), consistent with haploid maternal inheritance and destruction of the paternal NIC59 genome. Although 3/31 embryos had a heterozygous genotype (red stars), we found that UV-irradiation can fail to completely destroy the paternal genome (see S8B Fig), suggesting that these three heterozygotes may result from haploid maternal inheritance of a JU1825 allele and haploid paternal inheritance of a NIC59 allele due to incomplete destruction of paternal DNA. (B) The paternal genome is not always eliminated by UV irradiation. C. nouraguensis (JU1825) females were mated to UV-irradiated C. nouraguensis (NIC59) males. Dead embryos produced by this cross were PCR genotyped at a single autosomal locus (oPL78+79). The gel shows the genotypes of a fraction of the dead embryos assayed. Red stars indicate cases in which the UV-irradiated paternal NIC59 DNA can be detected. (PDF) [file pgen.1008520.s008.pdf]

S8 Fig

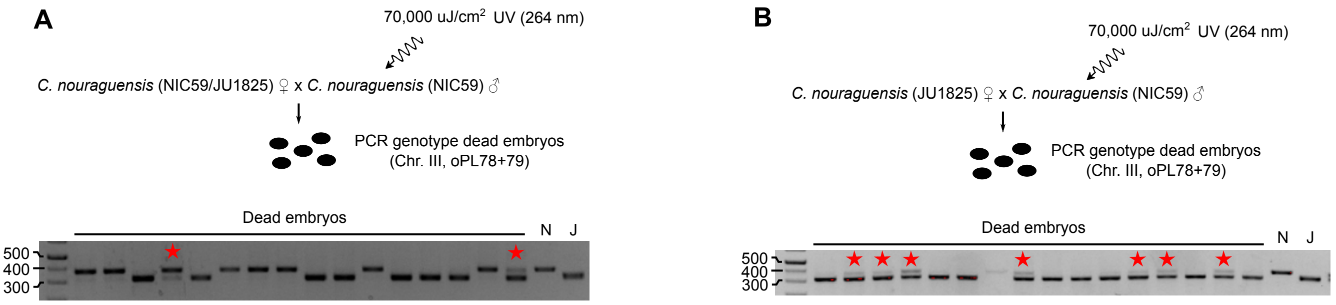

S8 Fig. Fertilization with UV-irradiated sperm does not cause frequent diploid maternal inheritance in intraspecies crosses. (A) Fertilization with UV-irradiated sperm does not significantly increase diploid maternal inheritance in *C. nouraguensis* intraspecies crosses. Heterozygous *C. nouraguensis* (NIC59/JU1825) females were mated to UV-irradiated *C. nouraguensis* (NIC59) males. Dead embryos produced by this cross were PCR genotyped at a single autosomal locus (oPL78+79). The gel shows the genotypes of a fraction of the dead embryos assayed. Most embryos had either a JU1825 (11/31) or NIC59 genotype (17/31), consistent with haploid maternal inheritance and destruction of the paternal NIC59 genome. Although 3/31 embryos had a heterozygous genotype (red stars), we found that UV-irradiation can fail to completely destroy the paternal genome (see Figure S8B), suggesting that these three heterozygotes may result from haploid maternal inheritance of a JU1825 allele and haploid paternal inheritance of a NIC59 allele due to incomplete destruction of paternal DNA. **(B)** The paternal genome is not always eliminated by UV irradiation. *C. nouraguensis* (JU1825) females were mated to UV-irradiated *C. nouraguensis* (NIC59) males. Dead embryos produced by this cross were PCR genotyped at a single autosomal locus (oPL78+79). The gel shows the genotypes of a fraction of the dead embryos assayed. Red stars indicate cases in which the UV-irradiated paternal NIC59 DNA can be detected.
