# Supplementary material for: Hybridization promotes asexual reproduction in Caenorhabditis nematodes
Source: PLoS Genet. 2019 Dec 16;15(12):e1008520. doi: 10.1371/journal.pgen.1008520 (PMC6946170; doi:10.1371/journal.pgen.1008520)
Supplement: S1 Table — Whole-genome amplified F1 individuals derived from the interspecies cross are labeled as "F1_" followed by a barcode number. Sample "F1_NIC59_JU1825" is one heterozygous NIC59/JU1825 female that was whole-genome amplified and serves as a control for genome-wide heterozygosity. Sample "NIC59plusJU1825" comprises one NIC59 and one JU1825 female placed in the same tube and whole-genome amplified. This also serves as a control for genome-wide heterozygosity. Sample "NIC59plusJU1825plusQG711" comprises one NIC59, one JU1825 and one QG711 female placed into the same tube and whole-genome amplified. This serves as a triploid (diploid C. nouraguensis and haploid C. becei) control. The "NIC59_bulk", "JU1825_bulk" and "QG711_bulk" samples are genomic preps made from large populations of each strain containing mixed developmental stages and sexes: these libraries were prepared without whole-genome amplification. The reads from the "NIC59_bulk" and "JU1825_bulk" samples were used to identify fixed SNPs between the two strains. The second column shows the interspecies cross that produced each viable F1 animal. In each case, heterozygous N/J C. nouraguensis females were crossed to C. becei QG711 males. However, the heterozygous C. nouraguensis females could have either a NIC59 or JU1825 mitochondrial genotype (denoted in parentheses). A fraction of each sample’s reads (Illumina 50-bp paired end) derive from E.coli (their food). The E. coli fraction is quite high in some samples, likely due to inefficient cleaning of single worms (see Materials and Methods). Of the reads that map to Caenorhabditis nuclear genomes, the approximate coverage and percent of all worm reads that map to each Caenorhabditis nuclear genome are shown. (PDF) [file pgen.1008520.s009.pdf]

						After filtering, number of reads matching						After filtering, percent of all assigned reads matching			After filtering, percent of all worm nuclear genome reads matching		Approximate coverage	
**Sample name**	Cross F1 derived from	F1 sex	F1 crossed to	F1 fertility	Total # reads	*E. coli*	Firmicutes	*C. nouraguensis* nuclear genome	*C. becei* nuclear genome	*C. nouraguensis* mitochondrial genome	*C. becei* mitochondrial genome	*E. coli*	*C. nouraguensis* nuclear genome	C. becei nuclear genome	*C. nouraguensis*	*C. becei*	*C. nouraguensis* nuclear genome	*C. becei* nuclear genome
**F1_1**	(N); N/J F1 female x QG711 male Plate 1	female	NIC59 male	fertile	18,405,750	11,551,300	19	3,529,806	10,854	9,412	103	76.5	23.4	0.1	99.7	0.3	2.4	0.0
**F1_5**	(N); N/J F1 female x QG711 male Plate 2	female	NIC59 male	fertile	4,709,920	306,730	6	3,291,061	6,828	5,605	61	8.5	91.2	0.2	99.8	0.2	2.2	0.0
**F1_8**	(N); N/J F1 female x QG711 male Plate 2	female	NIC59 male	fertile	6,219,960	2,499,294	4	2,528,892	5,174	4,160	48	49.6	50.2	0.1	99.8	0.2	1.7	0.0
**F1_11**	(N); N/J F1 female x QG711 male Plate 3	female	NIC59 male	fertile	5,556,004	1,228,558	3	3,169,866	6,505	1,879	20	27.9	71.9	0.1	99.8	0.2	2.2	0.0
**F1_25**	(J); N/J F1 female x QG711 male Plate 3	female	JU1825 male	fertile	6,489,980	5,089,384	3	369,912	8,714	499	8	93.1	6.8	0.2	97.7	2.3	0.3	0.0
**F1_29**	(J); N/J F1 female x QG711 male Plate 4	female	JU1825 male	fertile	7,097,772	667,344	13	4,884,750	9,066	1,495	16	12.0	87.8	0.2	99.8	0.2	3.3	0.0
**F1_39**	(J); N/J F1 female x QG711 male Plate 1	female	JU1825 male	fertile	4,858,964	688,291	5	3,186,443	7,190	3,346	14	17.7	82.0	0.2	99.8	0.2	2.2	0.0
**F1_41**	(J); N/J F1 female x QG711 male Plate 2	female	JU1825 male	fertile	18,011,610	1,112,932	20	12,756,921	21,799	2,298	19	8.0	91.8	0.2	99.8	0.2	8.7	0.0
**F1_4**	(N); N/J F1 female x QG711 male Plate 1	male	NIC59 female	fertile	13,552,874	151,066	6	10,195,147	22,476	5,451	51	1.5	98.3	0.2	99.8	0.2	7.0	0.0
**F1_46**	(N); N/J F1 female x QG711 male Plate 1	male	NIC59 female	fertile	20,496,550	4,494	15	15,463,159	35,591	7,759	83	0.0	99.7	0.2	99.8	0.2	10.6	0.0
**F1_48**	(N); N/J F1 female x QG711 male Plate 4	male	NIC59 female	fertile	5,484,366	13,691	3	4,151,082	9,759	661	6	0.3	99.4	0.2	99.8	0.2	2.8	0.0
**F1_6**	(N); N/J F1 female x QG711 male Plate 2	female	NIC59 male	sterile	4,477,900	930,700	7	1,932,322	705,081	1,202	15	26.1	54.1	19.8	73.3	26.7	1.3	0.4
**F1_12**	(N); N/J F1 female x QG711 male Plate 3	female	NIC59 male	sterile	7,884,868	753,139	7	3,978,335	1,483,331	2,085	19	12.1	64.0	23.9	72.8	27.2	2.7	0.8
**F1_18**	(J); N/J F1 female x QG711 male Plate 1	female	JU1825 male	sterile	3,468,706	1,496,149	3	1,373,037	7,652	96	0	52.0	47.7	0.3	99.5	0.6	0.9	0.0
**F1_20**	(J); N/J F1 female x QG711 male Plate 2	female	JU1825 male	sterile	10,834,856	1,163,152	16	5,203,891	1,822,729	4,026	23	14.2	63.5	22.2	74.1	25.9	3.6	1.0
**F1_26**	(J); N/J F1 female x QG711 male Plate 3	female	JU1825 male	sterile	3,594,908	891,072	3	1,923,182	92,400	690	4	30.6	66.1	3.2	95.4	4.6	1.3	0.0
**F1_10**	(N); N/J F1 female x QG711 male Plate 2	male	NIC59 female	sterile	13,848,208	400,238	7	8,508,436	1,589,956	1,200	9	3.8	81.0	15.1	84.3	15.7	5.8	0.9
**F1_16**	(N); N/J F1 female x QG711 male Plate 3	male	NIC59 female	sterile	15,639,042	288,535	2	10,408,444	1,046,790	16,142	172	2.5	88.5	8.9	90.9	9.1	7.1	0.6
**F1_17**	(N); N/J F1 female x QG711 male Plate 3	male	NIC59 female	sterile	6,247,186	79,293	15	3,443,948	1,272,137	322	9	1.7	71.8	26.5	73.0	27.0	2.4	0.7
**F1_21**	(J); N/J F1 female x QG711 male Plate 2	male	JU1825 female	sterile	9,834,614	137,887	6	5,701,336	1,741,990	480	5	1.8	75.2	23.0	76.6	23.4	3.9	0.9
**F1_23**	(J); N/J F1 female x QG711 male Plate 2	male	JU1825 female	sterile	12,059,396	1,570,440	6	7,254,493	382,635	11,575	96	17.0	78.7	4.2	95.0	5.0	5.0	0.2
**F1_NIC59_JU1825**	JU1825 female x NIC59 male	female			8,237,250	75,877	14	6,344,331	12,349	3,180	38	1.2	98.6	0.2	99.8	0.2	4.3	0.0
**NIC59plusJU1825**		female			7,045,822	319,354	15	5,091,335	10,238	2,668	21	5.9	93.9	0.2	99.8	0.2	3.5	0.0
**NIC59plusJU1825plusQG711**		female			11,160,274	275,223	7	5,895,338	2,620,526	1,864	620	3.1	67.0	29.8	69.2	30.8	4.0	1.4
**NIC59_bulk**		mixed			32,250,274	257,304	31	22,649,102	52,717	50,912	437	1.1	98.4	0.2	99.8	0.2	15.5	0.0
**JU1825_bulk**		mixed			36,926,106	357,701	394	27,761,970	44,405	58,749	279	1.3	98.4	0.2	99.8	0.2	19.0	0.0
**QG711_bulk**		mixed			28,828,734	235,533	37	120,477	21,138,043	17	41,249	1.1	0.6	98.2	0.6	99.4	0.1	11.4

S1 Table. Characteristics of the samples submitted for whole-genome sequencing. Whole-genome amplified F1 individuals derived from the interspecies cross are labeled as "F1_" followed by a barcode number. Sample "F1_NIC59_JU1825" is one heterozygous NIC59/JU1825 female that was whole-genome amplified and serves as a control for genome-wide heterozygosity. Sample "NIC59plusJU1825" comprises one NIC59 and one JU1825 female placed in the same tube and whole-genome amplified. This also serves as a control for genome-wide heterozygosity. Sample "NIC59plusJU1825plusQG711" comprises one NIC59, one JU1825 and one QG711 female placed into the same tube and whole-genome amplified. This serves as a triploid (diploid *C. nouraguensis* and haploid *C. becei*) control. The "NIC59_bulk", "JU1825_bulk" and "QG711_bulk" samples are genomic preps made from large populations of each strain containing mixed developmental stages and sexes: these libraries were prepared without whole-genome amplification. The reads from the "NIC59_bulk" and "JU1825_bulk" samples were used to identify fixed SNPs between the two strains. The second column shows the interspecies cross that produced each viable F1 animal. In each case, heterozygous N/J *C. nouraguensis* females were crossed to *C. becei* QG711 males. However, the heterozygous *C. nouraguensis* females could have either a NIC59 or JU1825 mitochondrial genotype (denoted in parentheses). A fraction of each sample’s reads (Illumina 50-bp paired end) derive from *E.coli* (their food). The *E. coli* fraction is quite high in some samples, likely due to inefficient cleaning of single worms (see Materials and Methods). Of the reads that map to *Caenorhabditis* nuclear genomes, the approximate coverage and percent of all worm reads that map to each *Caenorhabditis* nuclear genome are shown.
